# Modelling microclimatic variability in Andean forests of northern Patagonia

**DOI:** 10.1007/s00484-025-02891-x

**Published:** 2025-03-25

**Authors:** Jonas Fierke, Birgitta Putzenlechner, Alois Simon, Juan Haridis Gowda, Ernesto Juan Reiter, Helge Walentowski, Martin Kappas

**Affiliations:** 1 Institute of Geography, University of Goettingen, Goldschmidtstraße 3, 37077 Goettingen, Germany; 2 Faculty of Resource Management, University of Applied Science and Art, Daimlerstraße 2, 37075 Goettingen, Germany; 3 INIBIOMA, Universidad Nacional del Comahue-CONICET, Quintral 1250, Bariloche, 8400 Argentina; 4 Albrecht-von-Haller-Institute for Plant Science, University of Goettingen, Untere Karspüle 2, 37073 Goettingen, Germany

**Keywords:** Random forest-based regression, Remote sensing, Microclimate, Forests, Statistical downscaling

## Abstract

Information on microclimatic conditions beneath canopies is key to understanding small-scale ecological processes, especially concerning the response of biodiversity to climate change. In north-western Patagonia, where data on climate-driven species distribution are scarce, our study provides valuable insights by providing microclimatic models covering spatiotemporal dynamics at 30 × 30 m resolution. Applying in-situ data from 2022 to 2024, we employed a random forest-based regression to assess the impact of several biophysical predictor variables describing terrain and vegetation properties on four microclimatic response variables at three vertical levels within forests. We also interpolated this data spatiotemporally, using statistical downscaling of ERA5 data. Our analysis reveals that the influence of the predictor variables varies strongly by month and response variable. Moreover, significant variability was observed between the models and months regarding their explanatory power and error range. For instance, the model predicting maximum air temperature at a 2 m height achieved an R² of 0.88 and an RMSE of 1.5 °C, while the model for minimum air temperature resulted in an R² of 0.73 and an RMSE of 1.8 °C. Our model approach provides a benchmark for spatiotemporal projections in this data-scarce region, aligned with the climate normal from 1981 to 2010. Future refinement could benefit from data on snow cover, land use and land cover, soil, as well as structural information on vegetation over an extended period, to enhance the dynamical aspects of microclimatic modelling. We are confident that our present model will substantially enhance possibilities to analyse species distribution across vegetation types and terrain-related features within the area.

**Supplementary Information:**

The online version contains supplementary material available at 10.1007/s00484-025-02891-x.

## Introduction

Small-scale variations in temperature, humidity, and soil moisture significantly influence forest ecosystems, affecting biological and biochemical functions. These microclimatic conditions impact the distribution and persistence of understory species by influencing their physiological traits and adaptation strategies (Geiger et al. 2009). Additionally, microclimate drives nutrient cycling, notably through its effects on litter decomposition (Coûteaux et al. 1995) and soil organic matter turnover (Conant et al. 2011). The microclimate within a forest, such as the thermal environment, affects various aspects of understory plant dynamics, including growth rates, photosynthetic activity, and interspecific competition (Geiger et al. 2009; Vinod et al. 2023).

Forest microclimate results from interactions between terrain, vegetation, and macroclimate. Terrain affects factors like sunlight exposure and drainage, causing temperature and moisture variations in small areas (Barry 2008; Aalto et al. 2017). Vegetation, through canopy density and type, regulates solar radiation and transpiration, creating diverse conditions below (Hardwick et al. 2015; Barry and Blanken 2016). Macroclimate establishes broader environmental conditions that influence these microclimates at both regional and local scales (De Frenne et al. 2021). The resulting mosaic of microclimates shapes ecological processes and species distributions. Climate change will likely alter microclimatic buffering, highlighting the need for research to understand its effects on ecosystem resilience (Maclean et al. 2015; Lenoir et al. 2017; Zellweger et al. 2020; De Frenne et al. 2021; Lombaerde et al. 2022). Understanding these interactions is essential for managing habitats and refugia, thereby conserving biodiversity (Hylander et al. 2022; Kemppinen et al. 2024).

The landscape of microclimatic research is characterized by a variety of methods that, among others, include the development of microclimatic models (Zellweger et al. 2019a; Lembrechts et al. 2022; Zignol et al. 2023). As a review on the discipline shows, future microclimatic research should aim to achieve greater flexibility in spatial and temporal scaling (Kemppinen et al. 2024). This flexibility, in turn, can further help to represent abiotic conditions at scales that define the distribution and performance of species as well as ecosystem functions and services (Potter et al. 2013; Lembrechts et al. 2022). So far, an increasing number of studies are addressing this issue. For instance, Haesen et al. (2021) and Zignol et al. (2023) have implemented spatiotemporal interpolation techniques, utilizing ground-based measurements and geospatial data, to predict long-term microclimatic patterns in forests at continental and local scales. Lembrechts et al. (2020) are constantly improving their global database on soil and near-surface temperatures that has already demonstrated its function as valuable input data for spatiotemporal modelling, all the way up to a global scale (Lembrechts et al. 2022). Previous research has shown that microclimatic models substantially improve our understanding of spatial interactions between species and their environments at local levels (Zellweger et al. 2019a). Other research has also demonstrated that microclimate modelling benefits from remote sensing data integration, especially in landscapes where ground-based measurements are challenging (Zignol et al. 2023). However, as Klinges et al. (2024) point out, it is not always higher spatial or temporal resolution that improves the prediction accuracy of ecological responses. Rather, it requires datasets with high climate proximity, i.e., datasets that represent the climatic conditions that organisms under consideration are exposed to. Collectively, these arguments underscore the critical role of advanced microclimatic mapping and modelling techniques in enhancing our predictive capabilities for diverse ecosystems under different climatic conditions.

Despite progress in microclimatic modelling, some regions remain underrepresented, including northern Patagonia, where gridded microclimatic data is still lacking. This is further reinforced by the considerable uncertainties within the region’s available high-resolution gridded climate datasets, which are crucial for ecological studies (Fierke et al. 2024). Moreover, these datasets have the disadvantage of not accurately representing the climatic conditions experienced by organisms under the canopy and underneath the surface, focusing instead on open-air temperatures (Fick and Hijmans 2017; Karger et al. 2017). As a result, critical processes, such as how species reliant on stable microclimates respond to climate change, remain poorly understood. Understanding these processes is essential, as climate change poses a significant threat to suitable habitats for many plant species in northern Patagonia, potentially jeopardizing biodiversity hotspots and disrupting ecosystem stability (Soliani et al. 2024). While existing studies on microclimate in the region focus on the important issue of understanding microclimatic processes (Barberá et al. 2023; Simon et al. 2024), they do not aim to comprehensively create microclimatic models over a longer period and larger spatial extent. As a result, comprehensive spatial data is not yet available.

Given the absence of gridded microclimate data in northern Patagonia and the need for greater spatial and temporal coverage, as well as closer proximity in microclimatic modelling, our study aims to comprehensively analyse and project the microclimates of Andean forests in northern Patagonia. We address both historical changes and vertical variations within the constraints of existing data. Our objectives are to: (1) model the current microclimates of Andean forests in northern Patagonia and identify statistical relationships between predictors and microclimatic variables, (2) project these findings onto the historical period from 1981 to 2010 to assess past changes, (3) investigate vertical microclimatic variations at 6 cm depth and heights of 15 cm and 2 m, and (4) evaluate data limitations in northern Patagonia’s microclimatic modelling. Our analysis is based on measurements of three key microclimatic variables at different heights: air temperature at 2 m and 15 cm above ground and 6 cm below ground, relative humidity at 2 m, and volumetric water content (VWC) at 6 cm depth. Using a random forest regression model, we incorporate microclimatic observations from 2022 to 2024 alongside vegetation and terrain data from satellite imagery and local climate data from weather stations and downscaled ERA5 estimates. This approach enables spatial interpolation at 30-meter resolution and temporal interpolation for the historical period of 1981–2010. The combination of variables at multiple heights and depths allows us to assess initial vertical patterns within the microclimate. Finally, we analyse the current data used in our microclimatic predictions to identify which parts of the model suffer from data shortcomings and to understand the uncertainties these shortcomings cause. Our models, along with supplementary information, aim to characterize local microclimatic patterns and variations, providing essential insights for further studies on ecological processes beneath the canopy.

## Data and methods

### Study area

**Fig. 1:**
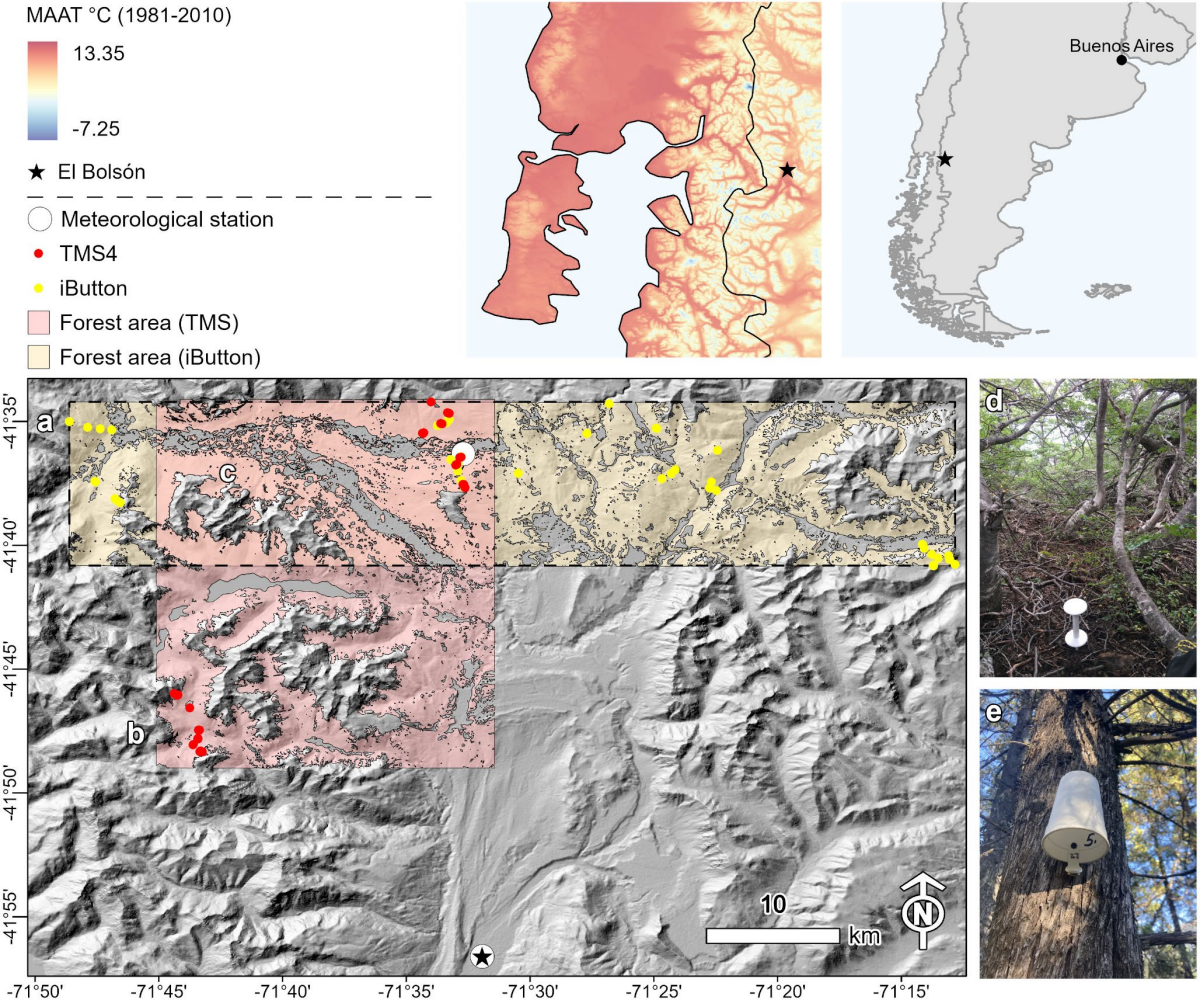
Study area. Where (**a**) is showing the section of iButton data loggers, (**b**) the section of TMS-4 data loggers, and (**c**) the intersection of both. Photos (**d**) and (**e**) show exemplary sites of TMS and iButton loggers respectively. Mean Annual Air Temperature (MAAT) of the region is based on climate data of Karger et al. (2017)

Our study focuses on two selected zones located to the north and west of El Bolsón in the Andes of northern Patagonia (Fig. 1). One zone, Fig. 1a, encompasses the Valle del Río Manso Inferior, approximately 50 km by 10 km in extent, while the other, Fig. 1b, includes parts of the Valle del Río Manso Inferior and areas around Lago Lahuán, measuring about 20 km by 30 km. Both zones overlap in a section covering parts of the Valle del Río Manso Inferior (Fig. 1c). The investigated landscape is predominantly covered by four native tree species: *Nothofagus pumilio* ((Poepp. & Endl.) Krasser), *N. antarctica* ((Forst.) Oerst.), *N. dombeyi* ((Mirb.) Oerst.), and *Austrocedrus chilensis* (D. Don) Pic.-Ser. & Bizz). The area features a macroclimate that is influenced by its geographical setting in valleys of various sizes within the Andes mountains. Furthermore, the area is characterized by two environmental key gradients: a pronounced precipitation gradient from west to east and an elevational gradient ranging from approximately 450 to 1,700 m. Annual precipitation recorded at the weather station in El Bolsón (1991–2020) at 345 m elevation averages 894 mm, ranging from 30 mm in February to 170 mm in June. The mean annual temperature is 10.3 °C, with the highest monthly mean temperature of 17.7 °C in February and the lowest of 3.8 °C in July (SMN 2024).

### Microclimatic data

Using TOMST TMS-4 Standard dataloggers (Wild et al. 2019), we recorded temperature at 15 cm height and both temperature and VWC at a soil depth of 6 cm below ground (Fig. 1d) every 15 min across 8 locations (20 loggers) from March 2022 to February 2024, and 8 locations (18 loggers) from March 2023 to February 2024. The dataloggers were installed across forest stands covering an elevational gradient from 600 to 1600 m (Fig. 1b) with replicates between 600 and 1400 m and individual loggers at the treeline between 1500 m and 1600 m, respectively. Additional temperature and relative humidity data, recorded at 4-hour intervals (02:00, 06:00, 10:00, 14:00, 18:00, and 22:00 UTC-3) from loggers at 2 m height (Fig. 1e), were collected from 40 sites, partially overlapping with the TMS loggers (Fig. 1a). This data spans March 2022 to February 2023 Simon et al. (2024) using iButton dataloggers (DS1923 Hygrochron) (Analog Devices, Inc., USA 2023). To ensure reliable measurements, the sensors were shielded from direct sunlight and adverse weather conditions, including precipitation and strong winds, using a white, ventilated enclosure to minimize environmental impact (Fig. 1e). All logger data underwent quality checks to remove errors and duplicates. For VWC, we only used daily records where the average soil temperature exceeded 0 °C, excluding 1.6% of days to eliminate frozen soil data (Wild et al. 2019). VWC was estimated using a simplified calibration based on Jačka et al. (2023), focusing on proportional trends rather than absolute values. Our calibration can be considered simplified because it is based on a single soil sample and average physical properties (e.g. bulk density) according to Simon et al. (in prep.). Calibration details appear in Figure S1, with logger time series shown in Figures S2–S4. We selected soil moisture, air humidity, and soil-, surface-, and air temperature as key variables for this study due to their fundamental roles in shaping microclimatic conditions relevant to organisms and ecological processes. These variables influence critical processes such as water availability, evapotranspiration, and thermal regulation, which are essential for shaping species distributions, physiological traits, and ecosystem functions (Barry 2008; Geiger et al. 2009; Conant et al. 2011; De Frenne et al. 2021). Furthermore, their widespread use in microclimatic studies (e.g., De Frenne et al. 2021; Kašpar et al. 2021; Aalto et al. 2022; Lembrechts et al. 2022) facilitates direct comparisons with existing datasets, enhancing the broader applicability of our findings.

### Macroclimatic data

We retrieved macroclimatic temperature data from a weather station located in the Valle del Río Manso Inferior at -41.61° South and − 71.54° West, and macroclimatic precipitation and humidity data from a weather station located in El Bolsón at -41.94° South and − 71.53° West. The Valle del Río Manso Inferior station has records from May 2003 to the present, with a gap between 2015 and 2019, while the El Bolsón station has records from January 1992 to the present (SMN 2024). While the station variables were directly integrated into the model calibration for the period from 2022 to 2024, we used ECMWF ERA5 predictors (Hersbach et al. 2020) for statistical downscaling (Table 1) to produce macroclimatic variables for the climate normal from 1981 to 2010. These variables measure various atmospheric conditions at different heights and pressure levels, including wind speed, wind direction, geopotential energy, humidity, temperature, and precipitation, essential for understanding and predicting weather patterns (Table S1). The variables were downloaded on a daily basis for the historical period from 1979 to 2014, as provided by the Canadian Centre for Climate Modelling and Analysis (ECCC 2024). By focusing on the historical period 1981–2010, future studies could compare the results of our research with existing models, such as those developed by Karger et al. (2017).

**Table 1:** Predictor variables for statistical downscaling of ERA5 data. Using even years from 2004 to 2014 for the Valle Del Río Manso inferior station and even years from 1991 to 2014 for the El Bolsón station, predictor variables were selected from the 26 available options (Table S1) based on their partial correlation coefficients (r) and p-values. Where hPa is the atmospheric pressure level in hectopascals

Variable (Station)	No.	ERA5 predictor variable	Partial r	*P* value
TMAX: maximum daily temperature (Valle del Río Manso Inferior)	1	Mean sea level pressure	-0.242	< 0.0001
	2	500 hPa Wind speed	-0.139	< 0.0001
	3	500 hPa Meridional wind component	0.184	< 0.0001
	4	500 hPa Geopotential	0.235	< 0.0001
	5	850 hPa Zonal wind component	-0.120	< 0.0001
	6	850 hPa Geopotential	0.132	< 0.0001
TMIN: minimum daily temperature (Valle del Río Manso Inferior)	1	Mean sea level pressure	-0.195	< 0.0001
	2	1000 hPa Wind speed	0.261	< 0.0001
	3	1000 hPa Relative vorticity of true wind	0.190	< 0.0001
	4	1000 hPa Divergence of true wind	-0.145	< 0.0001
	5	500 hPa Geopotential	0.290	< 0.0001
	6	Total precipitation	-0.112	< 0.0001
	7	850 hPa Specific humidity	0.313	< 0.0001
PRCP: daily sum of precipitation (El Bolsón)	1	1000 hPa Zonal wind component	-0.189	< 0.0001
	2	500 hPa Wind speed	0.112	< 0.0001
	3	850 hPa Wind Speed	0.139	< 0.0001
	4	850 hPa Geopotential	-0.240	< 0.0001
	5	850 hPa Divergence of true wind	-0.107	< 0.0001
RH: relative humidity (El Bolsón)	1	1000 hPa Divergence of true wind	0.157	< 0.0001
	2	850 hPa Divergence of true wind	-0.292	< 0.0001
	3	850 hPa Specific humidity	0.532	< 0.0001
	4	Air temperature at 2 m	-0.667	< 0.0001

### Vegetation and terrain data

For vegetation-related variables, we used the Enhanced Vegetation Index (EVI) and Normalized Difference Moisture Index (NDMI) based on Landsat 8/9 (USGS 2024), as well as the Leaf Area Index (LAI) and Fraction of Photosynthetically Active Radiation (FAPAR) based upon Sentinel 3/OLCI data (Copernicus Service Information 2022). EVI and NDMI have a spatial resolution of 30 m, whereas LAI and FAPAR feature a resolution of 300 m. All four indices are calculated monthly, based on an iterative interpolation process, during which pixels from months with cloud cover are replaced by data from subsequent available months. For EVI and NDMI, pixels affected by clouds and cloud shadows are excluded, based on the quality assessment file. Additionally, LAI and FAPAR data are already masked to exclude cloud-affected areas. While LAI and FAPAR data are calculated using a ready-to-use product at 10-day intervals from 2022 to 2024, EVI and NDMI data are derived from 135 Landsat 8/9 scenes available between 2022 and 2024. For tree height, we used the product of Tolan et al. (2024) at a spatial resolution of 1 m. Additionally, we assessed forest cover by combining tree height with EVI, using thresholds of 3 m and 0.22, respectively. This approach, similar to that of (Zignol et al. 2023), helped establish the distance and direction to the forest edge with a resolution of 30 m. Our terrain-related variables included elevation, distance to sink, relative elevation, terrain roughness, the Topographic Position Index (TPI), the Topographic Wetness Index (TWI), and monthly solar radiation derived from SRTM data (Farr et al. 2007) with a resolution of approx. 25 m accessed through *elevatr* in RStudio (Hollister et al. 2023). Except for solar radiation (potential incoming solar radiation, PISR) which was calculated using *RSAGA* (Brenning et al. 2022), all other terrain-related variables were computed using *terra* in RStudio (Hijmans et al. 2024). Where necessary, data was resampled and reprojected to 30 × 30 m^2^ spatial resolution and the EPSG:32,719 coordinate system. For our model calibration, tree height data was retained at a 1 m spatial resolution to preserve its quality as a predictor variable. Detailed information on all variables and their processing can be found in Figure S5.

### Model calibration

**Fig. 2:**
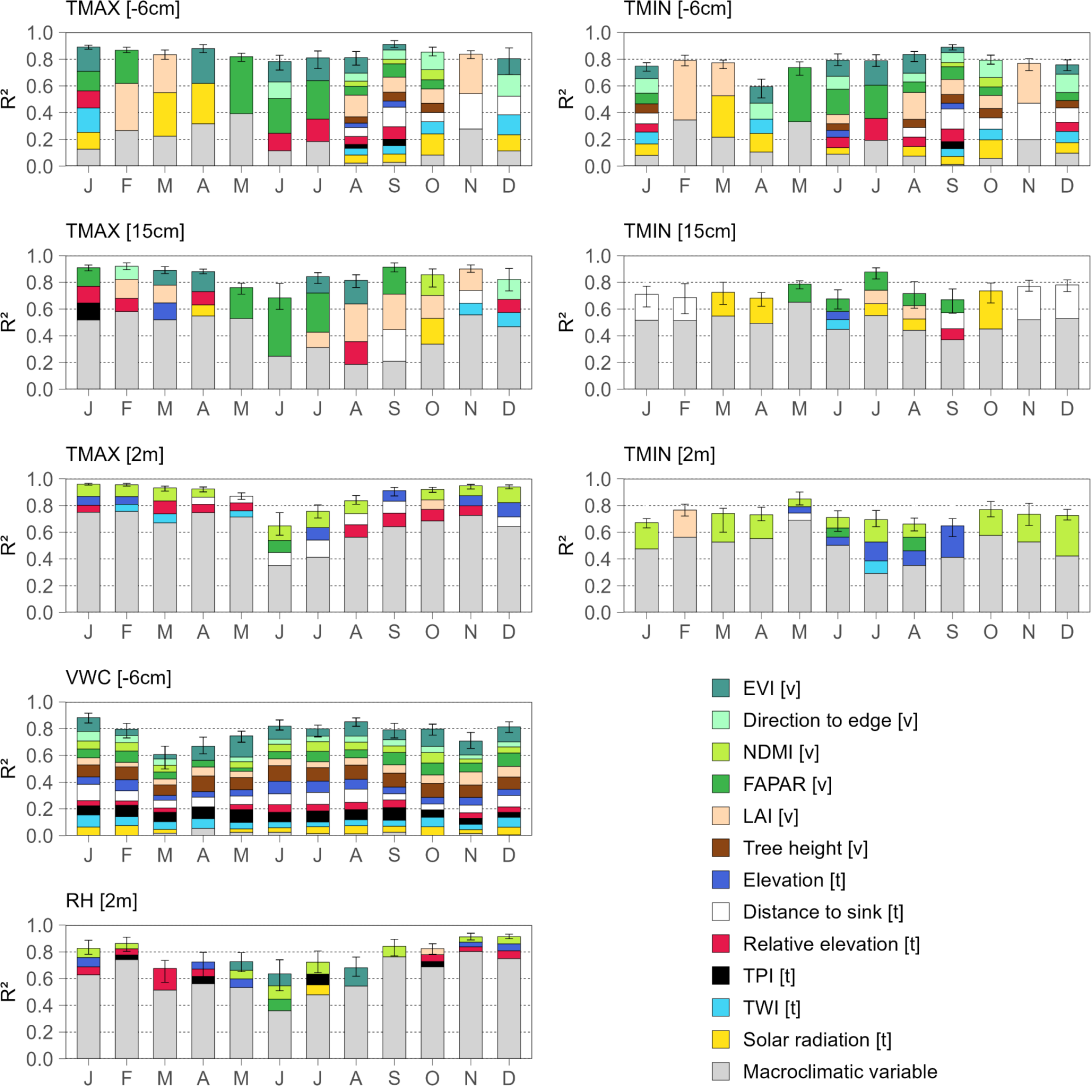
Relative importance of 13 selected biophysical variables for predicting microclimatic observations. Predictors are categorized as related to vegetation (v), topography (t), or macroclimate. The vertical lines in the bar indicate the range (maximum and minimum), while the bars represent the mean R² values across 100 ensembles. Each monthly bar corresponds to a specific set of biophysical variables identified after a recursive variable elimination process

We calibrated 8 models using monthly averages of the following observed microclimatic data as response variables: minimum and maximum daily temperatures at 6 cm depth, 15 cm and 2 m height, relative humidity at a height of 2 m, and VWC at a depth of 6 cm. These monthly averages were calculated by averaging the daily minimum and maximum values for each month. This approach was necessitated by the temporal resolution of the predictor variables, which were limited to monthly data. By averaging daily extremes over a month, we aligned the temporal resolution of the response variables with that of the predictors, ensuring consistency across the modelling framework. This method retains the critical information conveyed by extreme values while facilitating robust temporal interpolation. Before fitting the models, we selected 15 biophysical variables (related to vegetation, terrain, and macroclimate) as predictors: EVI, NDMI, LAI, FAPAR, tree height, distance to forest edge, direction to forest edge, elevation, relative elevation, terrain roughness, TPI, TWI, distance to sink, solar radiation, and macroclimate. The macroclimate variables at the meteorological station were customized according to the specific response variable being studied. Maximum and minimum daily temperature records were used for temperature-related variables, mean daily humidity records for humidity, and daily sum of precipitation data for VWC. We excluded collinear predictors by removing one predictor from each pair that exhibited a significant Pearson correlation coefficient greater than ± 0.7. This approach led to a removal of terrain roughness and distance to forest edge, as shown in Figure S6. To determine the best fit of variables, we applied recursive feature elimination based on a 10-fold cross-validation to each model and each month. This method helped us eliminate those variables that did not contribute additional explanatory power, measured by R², and reduced discrepancies between predicted and observed values, as indicated by the root mean square error (RMSE). To improve model robustness and assess predictive accuracy, we used a combination of 100 ensembles for each monthly model, where each ensemble consists of a randomly selected training and evaluation subset, comprising 80% and 20% of the data, respectively. This yielded us the variance of the individual model performance shown in Fig. 2. In total, our study design led to a processing of 96 cases from a combination of 8 models with reference to the observed microclimatic variables and 12 months.

All calculations were done using the *ranger* function in RStudio (Wright and Ziegler 2017), which applies the algorithm of Breiman (2001). The application of a random forest-based algorithm is based on the assumption that non-linear relationships between predictor and response variables exist (Chen et al. 1999; Zellweger et al. 2019b).

We performed spatiotemporal interpolation on our calibrated models and therefore utilized the Statistical DownScaling Model (SDSM) v.6.1, developed by Wilby et al. (2002) and Wilby and Dawson (2013) to fill data gaps in the weather station records and create a daily time series from 1981 to 2010 based on selected ERA5 predictor variables for statistical downscaling (Table S2). All ERA5 predictands used for downscaling station variables were selected based on their influence, assessed by partial correlation coefficients (r) and p-values, using the *screen variables* function in the SDSM software. For the recorded periods from 2004 to 2014 at Valle del Río Manso Inferior, and from 1992 to 2014 at El Bolsón respectively, even years were used as training data, while odd years served as validation data (cf. Zignol et al. 2023). Due to insufficient historical precipitation and humidity records at the meteorological station of Valle del Río Manso Inferior, the El Bolsón station was used as a substitute for downscaling these two variables. For each day of the synthesized time series, we generated 100 ensembles using the *weather generator* function and calculated the mean. Once the time series for minimum and maximum daily temperature, as well as mean daily humidity and daily sum of precipitation, were completed, these variables, along with the previously mentioned biophysical variables, served as predictors for temporally interpolating microclimatic variables from 1981 to 2010. Subsequently, we calculated monthly averages from these time series. These averages were then used for spatial interpolation to model microclimatic variables across different zones of our study area. The observation periods covered were 2022–2023 for iButton loggers and 2022–2024 for TMS loggers, in addition to a historical period from 1981 to 2010. Where one zone was established as an envelope encompassing all iButton loggers (Fig. 1a), and the other zone by an envelope of all TMS-4 loggers (Fig. 1b). Spatial interpolation was performed using the *predict* function in terra (Hijmans et al. 2024). Macroclimatic variables of the meteorological stations served as constant variables.

## Results

### Model calibration

As shown in Fig. 2, there is remarkable variability in the explanatory power and influence of predictors across the eight models. This variability can be observed among microclimatic variables (relative humidity, VWC, and minimum and maximum temperatures), their measurement scales (6 cm depth, 15 cm height, and 2 m height), and across different months. While several variables are relevant for predicting monthly VWC at a depth of 6 cm (median of 13 predictors), minimum temperature at 6 cm depth (median of 9 predictors), and maximum temperature at 15 cm height (median of 7 predictors), the number of relevant predictors for minimum temperature at a height of 2 m (median of 2 predictors) and at 15 cm (median of 2 predictors) is relatively low. Table 2 presents the three strongest predictor variables for all months in the prediction of VWC at 6 cm depth, minimum and maximum temperatures at 6 cm depth, 15 cm height, and 2 m height, as well as relative humidity at 2 m height.

**Table 2:** Summary of top predictor variables for microclimatic response variables. Predictor variables are listed according to their relevance, expressed as percentages, across all months

Response Variable	No.	Predictor Variable	Importance (%)
VWC [-6 cm]	1	Tree height	14.1
	2	EVI	13.5
	3	TPI	10.3
TMIN [-6 cm]	1	LAI	26.4
	2	TMIN at station	19.7
	3	FAPAR	19.6
TMAX [-6 cm]	1	LAI	25.9
	2	FAPAR	24.1
	3	TMAX at station	21.3
TMIN [15 cm]	1	TMIN at station	68.3
	2	Distance to sink	27.0
	3	Solar radiation	22.6
TMAX [15 cm]	1	TMAX at station	48.9
	2	FAPAR	33.3
	3	Distance to sink	20.8
RH [2 m]	1	RH at station	77.9
	2	EVI	14.4
	3	FAPAR	13.7
TMIN [2 m]	1	TMIN station	67.2
	2	LAI	26.5
	3	NDMI	23.5
TMAX [2 m]	1	TMAX station	71.5
	2	FAPAR	13.9
	3	NDMI	10.9

Returning to Fig. 2, the models predicting maximum temperature demonstrate the best performance, with an R² of 0.88 at 2 m height, followed by 0.85 at 15 cm height, and 0.84 at 6 cm depth, averaged over all months. In contrast, the models predicting minimum temperature above the surface show the lowest performance, with an R² of 0.73 at both 2 m and 15 cm heights, averaged over all months. When considering individual months, R² for nearly all models decreases to 0.6 or below, and for the prediction of minimum temperature at a depth of 6 cm in May, it decreases to 0.5. Regarding the error rate of the models, measured by RMSE, the pattern is slightly different. The models predicting maximum temperature at 6 cm depth and 2 m height show RMSE values of 1.1 °C and 1.5 °C, respectively. Conversely, the models predicting minimum temperature at the same depth and height show RMSE values of 1.2 °C and 1.8 °C, respectively. However, at 15 cm height, this pattern is reversed, with an RMSE of 1.4 °C for the model predicting minimum temperature and an RMSE of 2.4 °C for the model predicting maximum temperature. Detailed statistics are provided in Table S3.

### Spatiotemporal interpolation

**Fig. 3:**
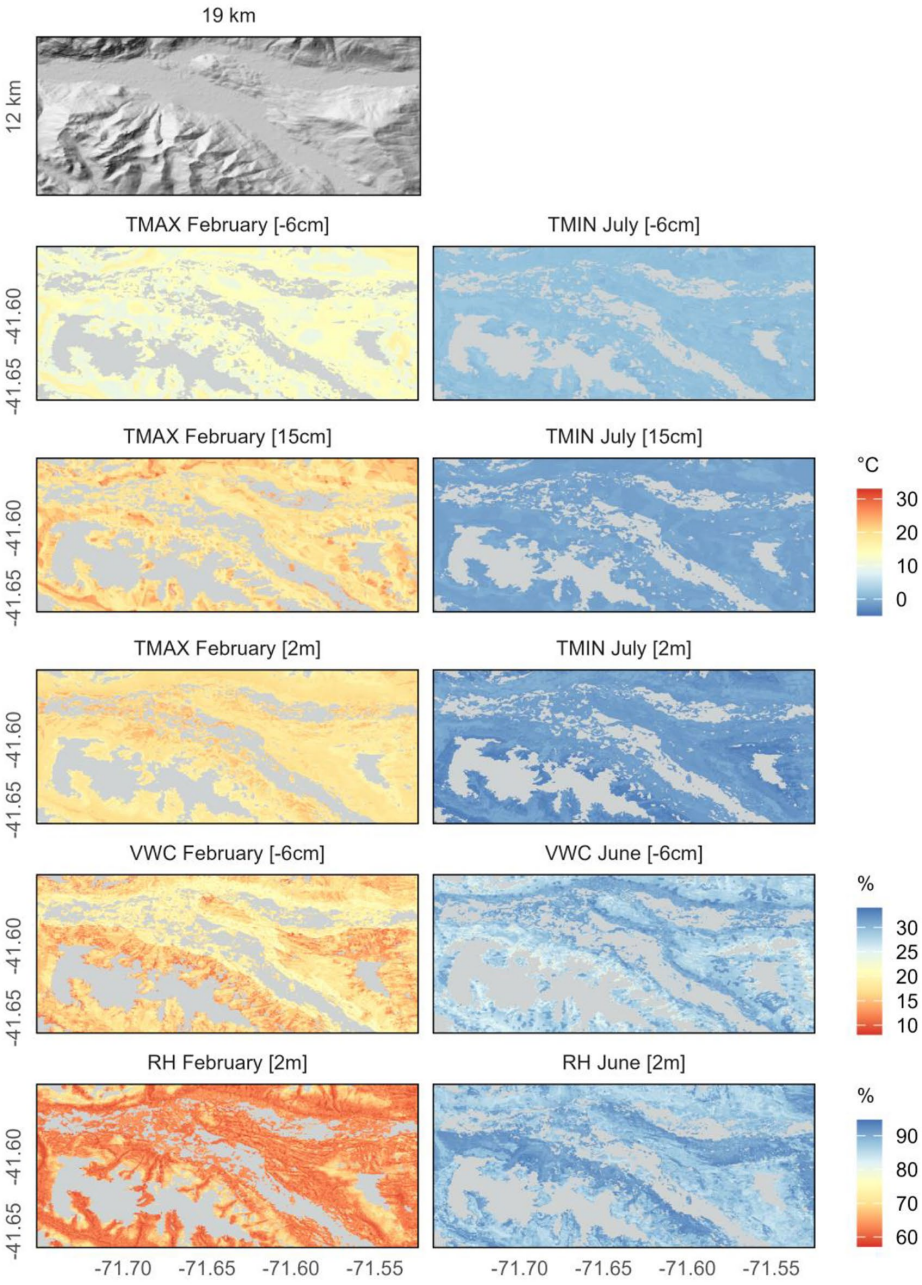
Selected examples of microclimatic maps in Valle del Río Manso Inferior (Fig. 1c). Depicted variables at a soil depth of 6 cm, as well as at heights of 15 cm and 2 m above ground level, for the period from 1981 to 2010. February represents the warm and dry extreme, July the cold extreme, and June the wet extreme. The section covers an area of 12 by 19 km and spans an elevational gradient from 433 to 2239 m. Grey areas indicate regions not covered by forest

The statistical downscaling of the ERA5 reanalysis data for daily temperature showed an R² of 0.69 and R² of 0.60 for maximum and minimum temperature respectively; and an R² of 0.28 and 0.35 for precipitation and humidity respectively (Figure S7). The results of our spatiotemporal interpolations were monthly models for the maximum and minimum temperature at 2 m height, 15 cm height, and 6 cm depth, as well as relative humidity at 2 m height and VWC at 6 cm depth. For illustrative presentation (Fig. 3), we selected three months of an exemplary section in Valle del Río Manso Inferior (Fig. 1c): February, as a dry and warm extreme, July as a cold extreme, and June as a wet extreme. All other months can be found in the appendix (Figure S8-S23).

### Comparison between historical and current microclimate

**Fig. 4:**
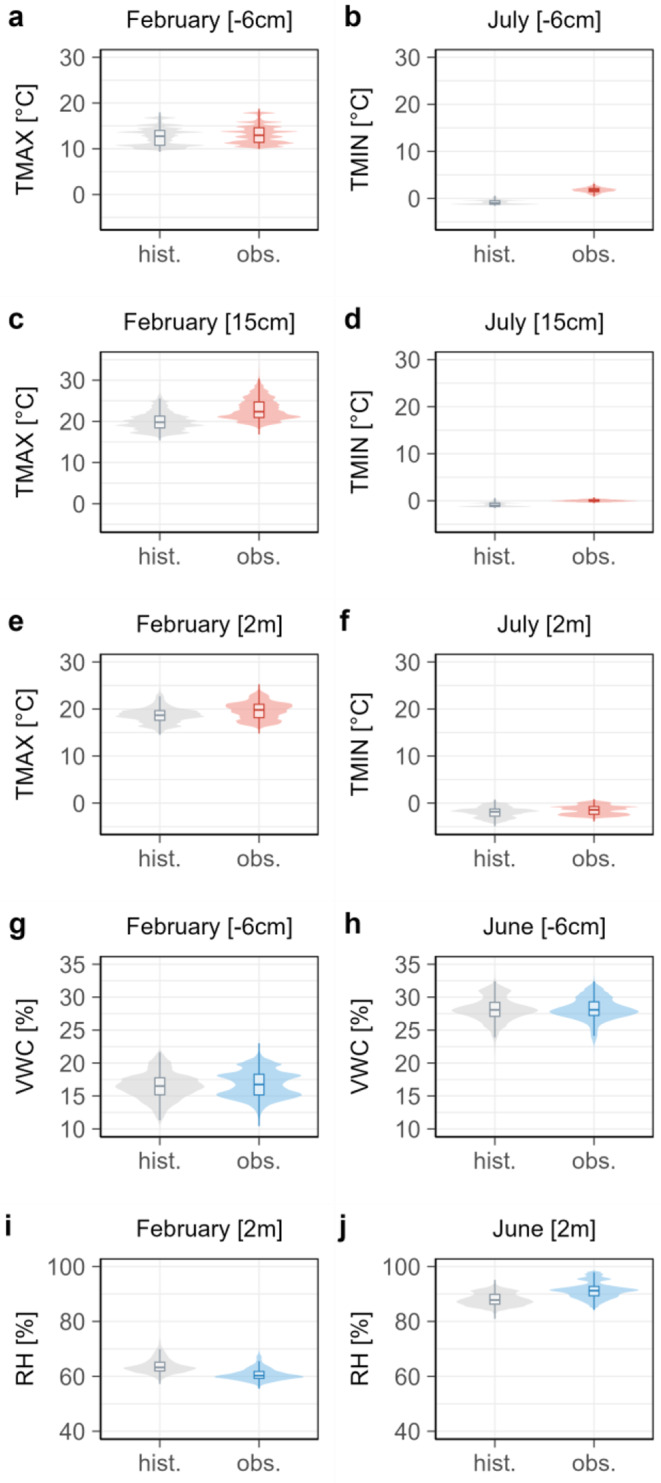
Microclimatic variables across periods and heights. Plots illustrating the distribution of microclimatic variables over historical and observed periods at 2 m and 15 cm heights, as well as 6 cm depth, highlighting the median, quartiles, and the frequency of values at different points. The observation period is 2022–2023 for the iButton loggers and 2022–2024 for the TMS loggers, while the historical reference period spans from 1981 to 2010. Temperature (red) at 6 cm depth and 15 cm height, as well as VWC (blue) at 6 cm depth, represent data from TMS loggers. Temperature (red) and relative humidity (blue) at 2 m height represent data from iButton loggers

Figure 4 compares microclimatic conditions depicted in our models of the historical period (1981–2010) with those from recent observation periods using iButton data (2022–2023) and TMS data (2022–2024). Most notably, temperatures at 6 cm below ground show a significant change in July’s minimum temperatures (Fig. 4, panel b), with the mean increasing from − 0.9 °C (SD of 0.4) to 1.8 °C (SD of 0.5), indicating fewer cold extremes. At 15 cm above ground, the strongest change is observed in maximum temperatures (Fig. 4, panel c), which rose from 20.1 °C (SD of 2.3) to 22.9 °C (SD of 2.6). In terms of VWC below ground, an increase in variance and range (Fig. 4, panel g) suggests greater variability in VWC during February over the observation period. At a height of 2 m, there is a slight increase in minimum temperatures from − 2.0 °C (SD of 1.1) to -1.5 °C (SD of 1.0) during July (Fig. 4, panel f). Further, maximum temperatures show an increase from 18.7 °C (SD of 1.7) to 19.7 °C (SD of 0.2) during February over the observation period. Regarding relative humidity at 2 m, an increase in variance and standard deviation indicates greater variability during June (Fig. 4, panel j). Besides these highlighted significant changes, the analysis did not reveal any other significant variations in the climate variables at depths of 6 cm and heights of 15 cm and 2 m between the historical reference period (1981–2010) and the observation period (2022–2024). It is important to consider the temperature accuracy of ± 0.5 °C within this comparative analysis (Wild et al. 2019).

### Vertical temperature variations

**Fig. 5:**
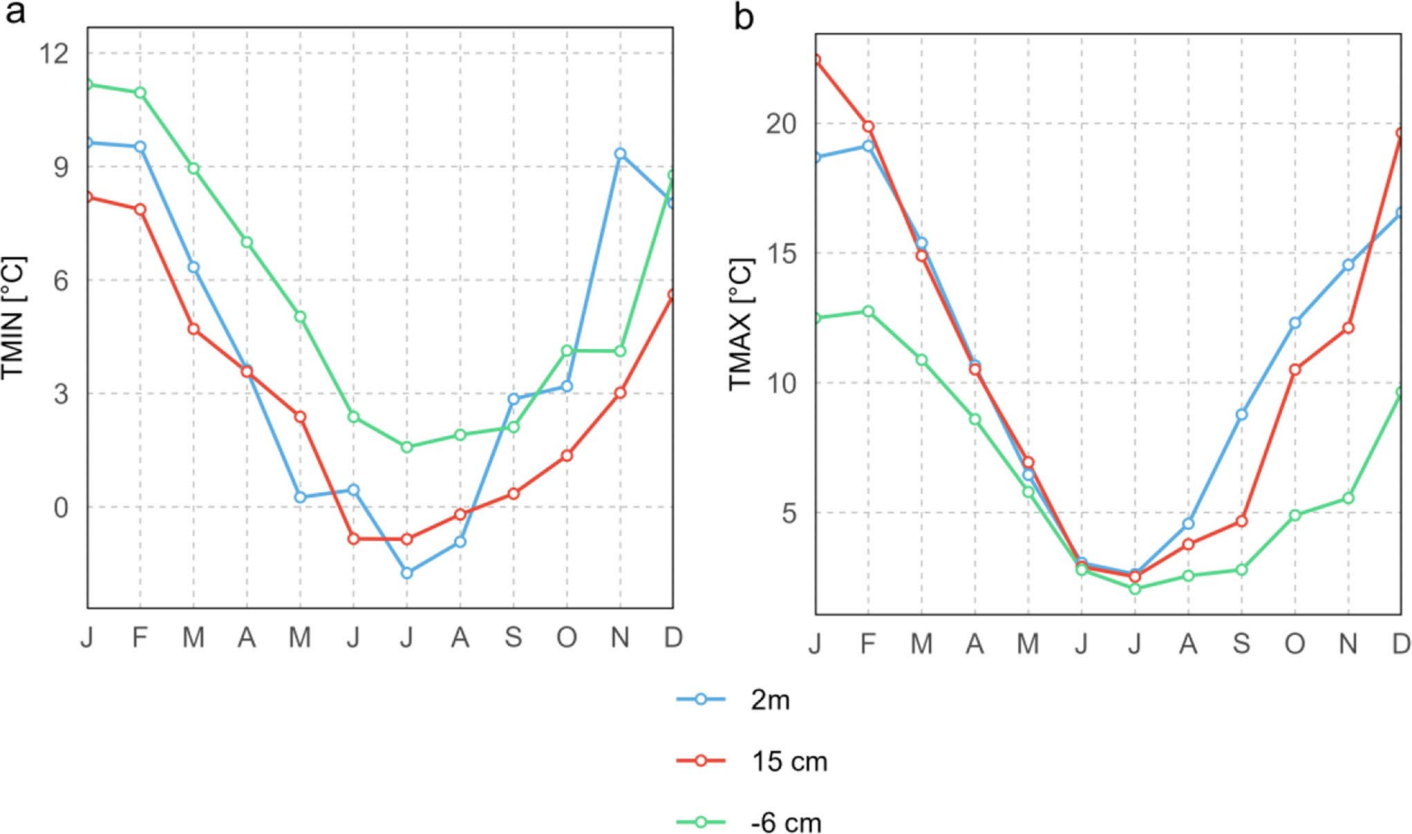
Vertical temperature variations. Where the left panel shows the mean monthly minimum and the right panel the mean monthly maximum temperatures for different vertical horizons at 2 m and 15 cm height, as well as 6 cm depth for the period from 1981 to 2010. Variations are related to a section of our study area where both spatial interpolations of iButton and TMS data overlap (Fig. 1c)

Figure 5 (panel a) shows the monthly course of mean daily minimum temperatures across three vertical horizons in the section of our study area where the interpolation of iButton data and TMS data overlaps (Fig. 1c). The minimum temperatures at a height of 15 cm are consistently lower than those at a depth of 6 cm, with an annual mean difference of 2.8 K. A similar pattern applies to the relation of minimum temperatures at a height of 2 m compared to those at a depth of 6 cm, though only until August. During September and November, minimum temperature at 6 cm depth exceeds that at 2 m height. The largest difference in minimum temperatures between two horizons occurs in November, with temperatures of 2.8 °C at 15 cm height and 9.6 °C at 2 m height, resulting in a difference of 6.7 K.

Figure 5 (panel b) illustrates the monthly course of mean daily maximum temperatures across the same vertical horizons and the same section. The highest maximum temperatures are observed at 15 cm height during December to February, reaching up to 23.6 °C. Intra-annual amplitude of maximum and minimum temperatures is more pronounced at heights of 2 m and 15 cm than those at a depth of 6 cm (Fig. 5 panel b). The largest differences in maximum temperatures between two horizons occur in January and December, with temperatures of 23.6 °C and 20.0 °C at 15 cm height, and 12.7 °C and 10.0 °C at 6 cm depth, resulting in differences of 10.8 K and 10.0 K, respectively.

## Discussion

We applied a random forest-based regression on microclimatic observations from 2022 to 2024, weather station records, and geospatial vegetation and terrain data to identify drivers of microclimatic variability in northern Patagonia. Additionally, we applied a statistical downscaling of ERA5 data to expand microclimatic models to a historical period (1981–2010). These models characterize local microclimatic patterns, forming a foundation for studies on understory vegetation and soil processes like respiration and nutrient cycling. Nevertheless, inherent uncertainties arise from limited biophysical data, sparse measurements, and the challenges of capturing dynamic ecological processes, as discussed later.

### Drivers of microclimatic variability

Our study shows that the statistical relationships between biophysical predictors and microclimatic variability varies significantly by microclimatic variable and season, a finding consistent with Aalto et al. (2022). We observed that predicting VWC at 6 cm depth required the most predictors (15), while estimating minimum temperature at 2 m height required the lowest (4). This discrepancy suggests that many predictors are not effective for estimating minimum temperature and humidity at 2 m. Fewer predictors can make models overly sensitive to those individual variables, leading to variability in our final models. One reason for the different number of predictors can be attributed to the variability of the underlying data. Specifically, lower variability in below-ground data tends to yield more reliable predictions. On the other hand, the high number of predictors needed to model underground temperature, WVC and winter RH, combined with their low consistency along the year, highlights the need for further exploring other predictors that better relate to these variables (Fig. 2).

For all models, except those focused on below-ground variables, local macroclimatic data, particularly air temperature and RH, exerted the most substantial influence. This result aligns with findings from Zignol et al. (2023), but suggests a slightly stronger macroclimate effect, with its influence ranging from 50 to 80%. This underscores the importance of establishing more local weather stations to capture detailed macroclimatic conditions in this region, which significantly impact microclimatic variations. Beyond macroclimatic drivers, solar radiation, FAPAR, NDMI, and distance to sink emerge as key predictors These factors influenced microclimate either during specific periods (e.g., FAPAR in winter temperature prediction at 15 cm depth) or consistently throughout the year (e.g., NDMI for 2 m temperature). This underscores that, alongside the potential incoming solar radiation, canopy structure and composition reflected through FAPAR, NDMI, and LAI are important factors of microclimatic regulation within forest ecosystems.

The overall explanatory power of monthly predictions and relative importance of predictors also varies substantially between months. This can be attributed to the seasonal climate of northern Patagonia and related changes in plant phenology, which is confirmed by another study in an environment with strong seasonal changes (Aalto et al. 2022). Winter data gaps also amplify seasonal effects, impacting model accuracy for winter months, a topic we will discuss in more detail below. The accuracies of our models also depend strongly on the microclimatic variables and months as it was also recognized in other studies (Lembrechts et al. 2022; Zignol et al. 2023). The highest accuracy was observed in the model predicting maximum temperature at a 2 m height (R² = 0.88, RMSE = 1.5 °C), while the lowest accuracy was in the model predicting minimum temperature at the same height (R² = 0.73, RMSE = 1.8 °C). In some months, the explanatory power dropped as low as R² = 0.6. These variations highlight the complexities of modelling microclimatic extremes at different heights, likely influenced by unrepresented predictors or those unavailable at an adequate scale.

### Microclimatic variability over space and time

Our models capture both landscape-specific variability and, to some extent, the vertical variability within 30-meter microclimate grid cells across our study area. At the landscape level, we observed over 10 K differences in maximum temperatures at 2 m height between exposed, sparsely forested areas at lower elevations and dense forests in higher elevations (e.g., Valle del Río Manso Inferior) during the warmest month from 2022 to 2023. Relative humidity also varied by up to 25% between these landforms in the same month. Such conditions likely impact tree species regeneration (Paritsis et al. 2015), species in the understory (Maclean et al. 2015; Morelli et al. 2016; Rahbek et al. 2019), and patterns of fuel moisture and fire disturbances (Barberá et al. 2023).

During summer (December to February), we recorded the highest maximum temperatures near the ground at 15 cm, while winter minimum temperatures were lower at 2 m than at 15 cm. This suggests that understory species encounter warmer maximum temperatures during the late growing season and less severe minimum temperatures in winter. Late winter and spring showed lower minimum and maximum temperatures at ground level compared to 2 m, contributing to extended winter conditions (Fig. 5). These conditions likely influence the vertical stratification of understory species (De Frenne et al. 2021; Aalto et al. 2022). The strongest vertical temperature difference occurred between 15 cm above ground and 6 cm below ground, with up to 10.8 K in January, indicating inefficient heat conduction into deeper soil. This significant temperature difference suggests that heat is not efficiently or quickly conducted into deeper soil layers. This could be attributed to the minerals soil’s coverage by an ecto-organic humus layer and understory vegetation, or to the generally low bulk density of volcanic ash soils, which inherently possesses lower thermal conductivity (Abu-Hamdeh and Reeder 2000; Buduba et al. 2020).

In addition to the spatial results of our study, our models capture current monthly microclimates from 2022 to 2024, as well as historical microclimates from 1981 to 2010, revealing microclimatic variability over time. We identified significant temperature increases at 15 cm and 2 m in recent years. The maximum temperatures increased from 20.1 °C to 22.9 °C at 15 cm, and from 18.7 °C to 19.7 °C at 2 m, indicating a notable rise in maximum temperatures over the observation period between 2022 and 2024. Subsurface minimum temperatures at 6 cm in July also increased from − 0.9 °C to 1.8 °C, indicating reduced cold extremes below ground. These temperature shifts are likely influenced by macroclimatic anomalies associated with mid-level anticyclonic patterns (Stella 2022, 2024; Collazo et al. 2024).

Our high-resolution spatial and temporal modelling provides critical insights for developing bioclimatic envelopes tailored to forest species and ecosystems. These results provide a framework for evaluating forest management strategies that consider both landscape and vertical climate variability, offering insights into species distribution patterns and understory dynamics, including tree regeneration (Kemppinen et al. 2024). Temporal interpolation of our models to 1981–2010 facilitates comparisons with other datasets from that period (cf. Karger et al. 2017) and supports the derivation of correction factors for macroclimatic models, as suggested by De Frenne et al. (2021).

### Data scarcity, model uncertainties, and prospects

A significant limitation of our model is its inability to capture intra-annual and multi-year dynamics due to missing or insufficient biophysical data. Key variables absent or only partially available include those related to vegetation, snow, soil properties, and human activities. Many vegetation-related variables, like tree height, are limited to single-year measurements, while cloud cover makes much winter data unusable, resulting in a bias towards capturing the seasonal changes of deciduous forests. Snow cover data, which is essential for understanding how near-surface temperature impacts soil moisture and overwintering organisms, is currently unavailable. This absence is significant, as snow’s insulating effect on soil temperatures plays a key role in the survival rates of these organisms (Williams et al. 2015).

Of 20,825 sensor-days analysed, 4,114 (about 19.8%) could have been affected by snow cover (adopting the method of Man et al. 2024), especially at higher altitudes and in winter, where model uncertainty is greatest. Variables like FAPAR and LAI, which provide canopy insights, significantly influence our models but could be enhanced with higher-resolution data, such as airborne LiDAR (Kašpar et al. 2021) or ground-based validation. This lack of dynamic vegetation data limits our ability to explain microclimatic variability across months and seasons, which we expect to impact future microclimates via shifts in disturbance regimes, land use, and species composition (Raffaele et al. 2011; Iglesias et al. 2022; Kitzberger et al. 2022).

Our sampling approach presents additional limitations. We used a random forest-based model, chosen for its ability to capture nonlinear relationships between biophysical predictors and microclimatic variables (Chen et al. 1999; Zellweger et al. 2019b). However, with few measurement points, there is an increased risk of overfitting. Future model enhancements could incorporate focal distance raster, which capture the influence of land cover within varying spatial buffers, to better reflect the broader environmental effects on microclimatic variability. This method, as demonstrated by Shandas et al. (2019), has been shown to improve model precision and predictive power by considering the spatial configuration of landscape features around measurement points. However, the inclusion would require substantial computational resources and processing. Regarding the existing VWC measurements, our study shows proportional trends rather than absolute values due to simplified calibration, potentially underrepresenting extreme conditions like droughts. This limitation is critical for ecological predictions.

Given these limitations, especially in environmental data quality and measurement scarcity, we recommend testing our gridded data at different spatial resolutions. Even at lower resolutions, our dataset can improve microclimatic representation beneath forest canopies in the northern Patagonian Andes. This refinement is crucial, as Klinges et al. (2024) have demonstrated that not only higher temporal and spatial resolution but particularly proximity, i.e., the change from macro- to microclimate, is essential for enhancing ecological models. Consequently, our datasets could at least enhance prior datasets by Karger et al. (2017) and Fick and Hijmans (2017), which primarily reflect open atmosphere conditions.

A further limitation is the use of VWC measurements at a shallow 6 cm depth, which raises concerns given the prevalence of summer droughts and deeper soil layers in our area. While 6 cm VWC data is valuable for understanding fuel moisture and wildfire risk (Jensen et al. 2018; Krueger et al. 2023), it may not represent the moisture levels experienced by plant roots in deeper soil. This could limit the effectiveness of these measurements in predicting plant water stress and ecosystem responses, suggesting the need for deeper VWC measurements. However, shallow VWC data does provide insight into the survival and growth of tree seedlings critical for forest regeneration (Caselli et al. 2019).

Addressing these limitations by incorporating dynamic data sources could enhance our models’ predictive accuracy, enabling more robust management strategies tailored to changing microclimates. Expanding the model to include disturbed areas could further inform forest regeneration processes and identify tipping points affecting vegetation structure. Simon et al. (2024) showed that microclimate varies significantly within shrub-like vegetation, yet our data does not cover all regional vegetation types. With disturbances projected to increase (Kitzberger et al. 2022), filling these gaps will be crucial for accurately projecting future microclimatic conditions.

Our findings on microclimatic variability provide a foundation for understanding how extreme temperature buffering can guide conservation and land management strategies in northern Patagonia. Identifying areas as potential climate refugia for vulnerable species highlights the critical role of microclimates in mitigating biodiversity loss under climate change (Beugnon et al. 2024; Kemppinen et al. 2024). Future efforts should prioritize integrating microclimatic processes into reforestation and land-use planning to enhance ecosystem resilience. By promoting vertical canopy complexity and minimizing soil exposure, management strategies can help buffer against extreme climatic events, support biodiversity conservation, and foster species adaptation. Additionally, fire risk assessments could be improved by incorporating microclimatic influences on fuel moisture and flammability. As demonstrated by Barberá et al. (2023), local microclimatic differences, such as cooler and moister conditions in forested areas compared to shrublands, significantly affect fuel moisture content and fire behaviour. Considering these microclimatic effects, alongside local precipitation, topography, and vegetation-related wind patterns, could enhance the accuracy of fire risk predictions and inform land management strategies. These actionable insights underscore the value of this work as a stepping stone for both scientific inquiry and practical applications in addressing climate challenges.

## Electronic supplementary material

Below is the link to the electronic supplementary material.

Supplementary Material 1

## Data Availability

Derived data supporting the findings of this study are available upon request from the corresponding author.
