# Supplementary material for: Modelling microclimatic variability in Andean forests of northern Patagonia
Source: Int J Biometeorol. 2025 Mar 25;69(6):1279–95. doi: 10.1007/s00484-025-02891-x (PMC12141355; doi:10.1007/s00484-025-02891-x)
Supplement: Supplementary file 1 — Supplementary Material 1 [file 484_2025_2891_MOESM1_ESM.docx]

# Supplementary Information


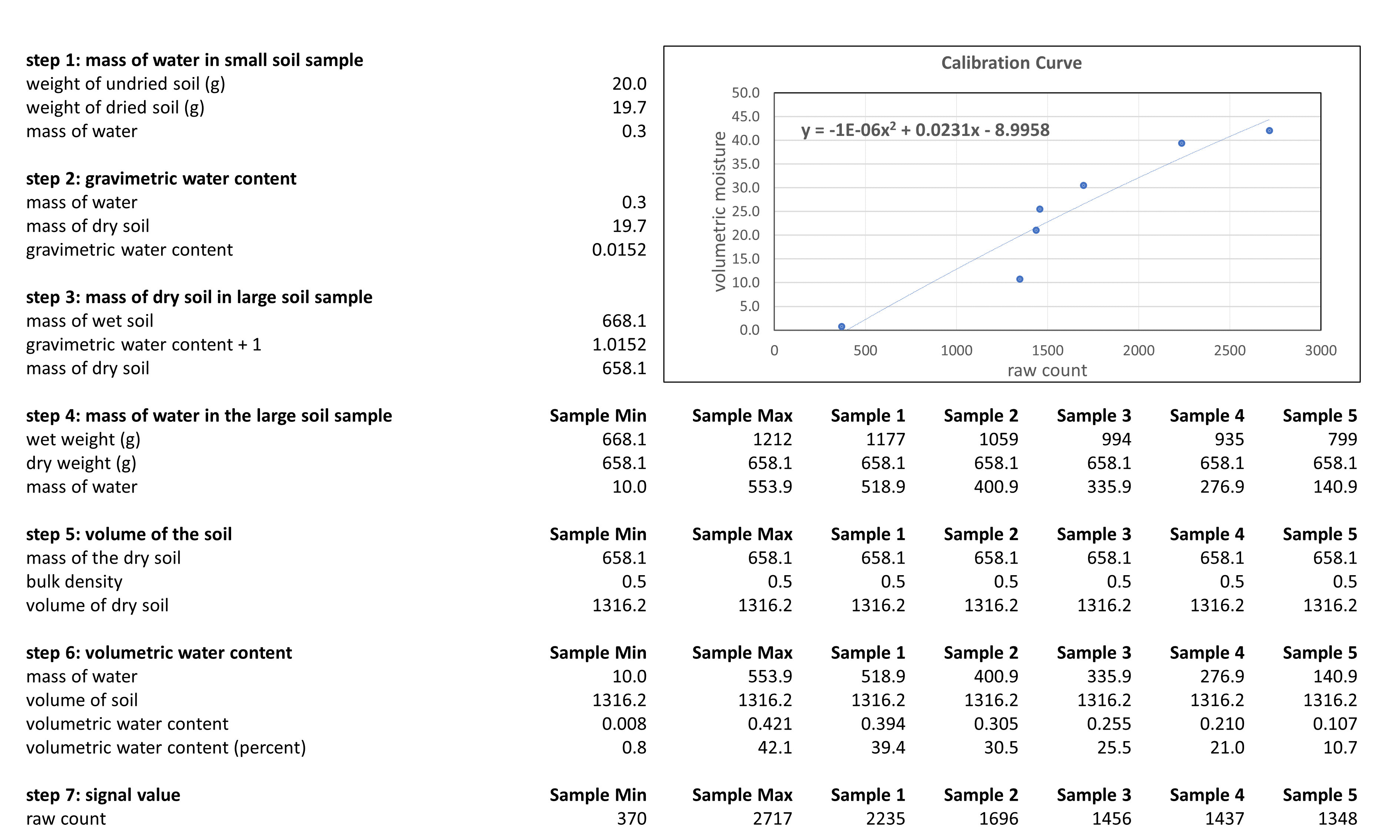


**Fig. 1** TMS Calibration. Individual steps were performed to establish the relationship between the raw signal values and volumetric water content. The result was fitted and plotted as a second-degree polynomial


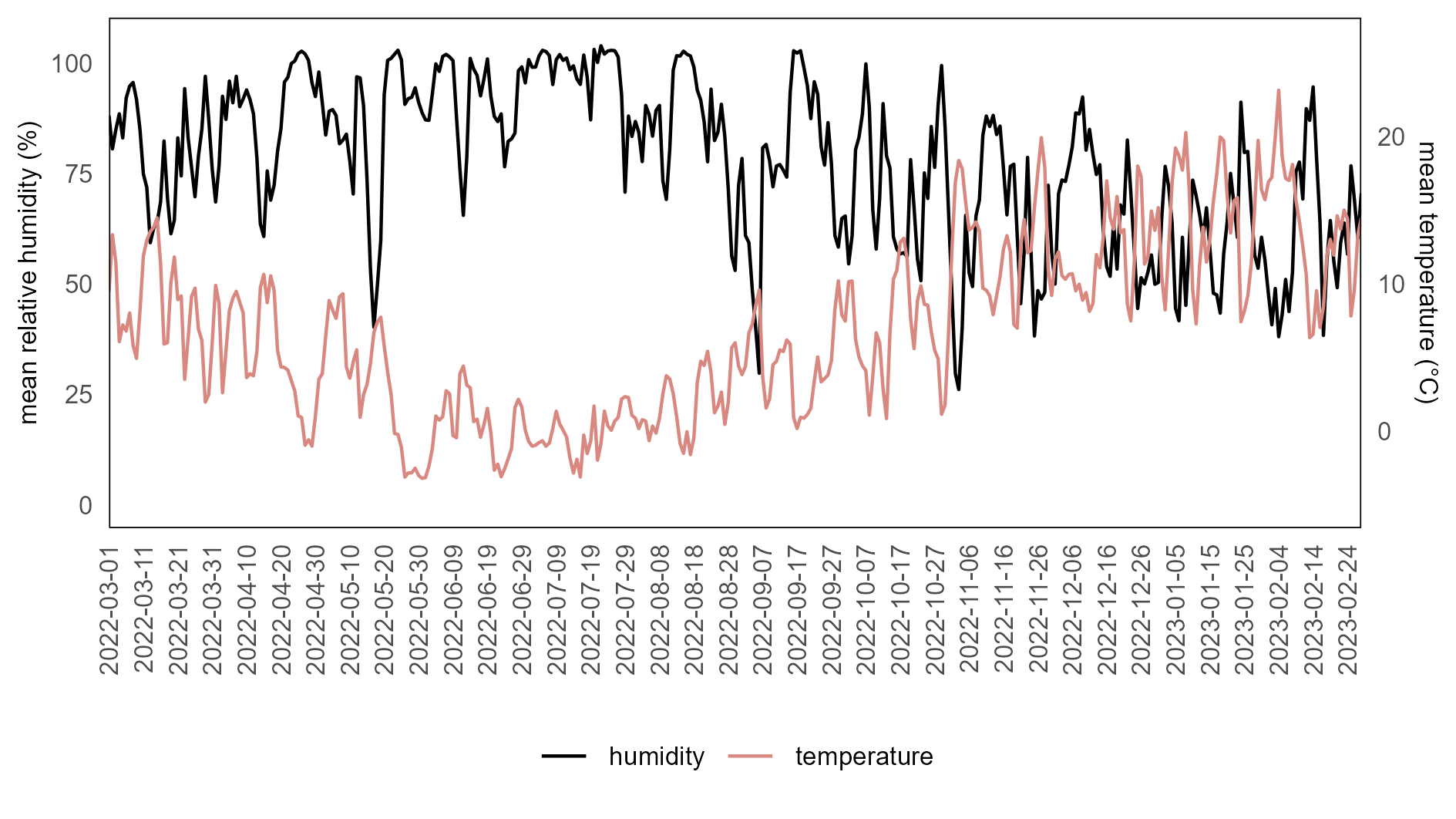


**Fig. 2** Mean daily temperature and mean daily humidity at 2m of all iButton loggers


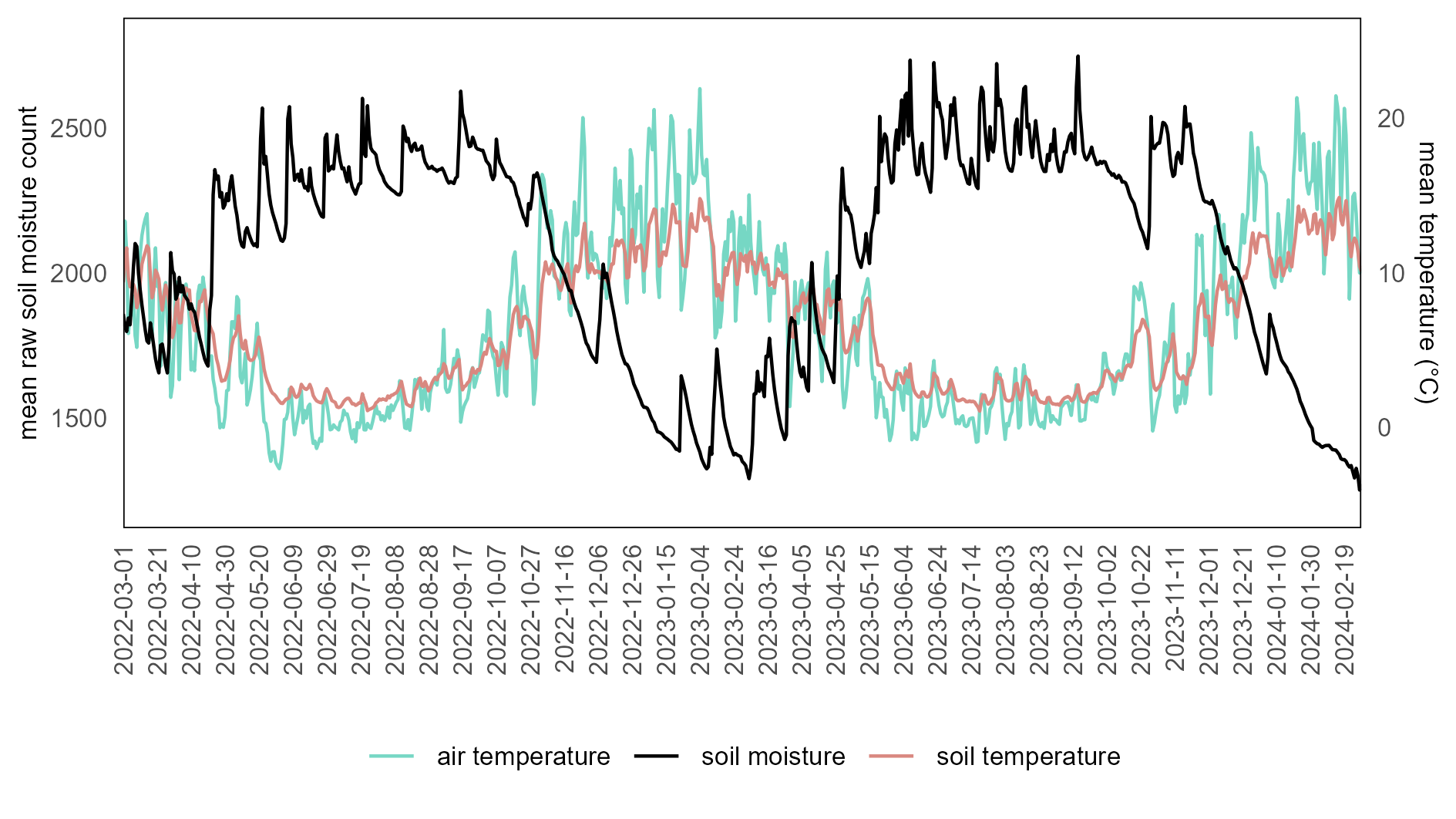


**Fig. 3** Mean daily temperature and mean daily raw soil moisture count from all TMS loggers, where soil moisture and soil temperature are measured at a depth of -6 cm, and air temperature is measured at a height of 15 cm


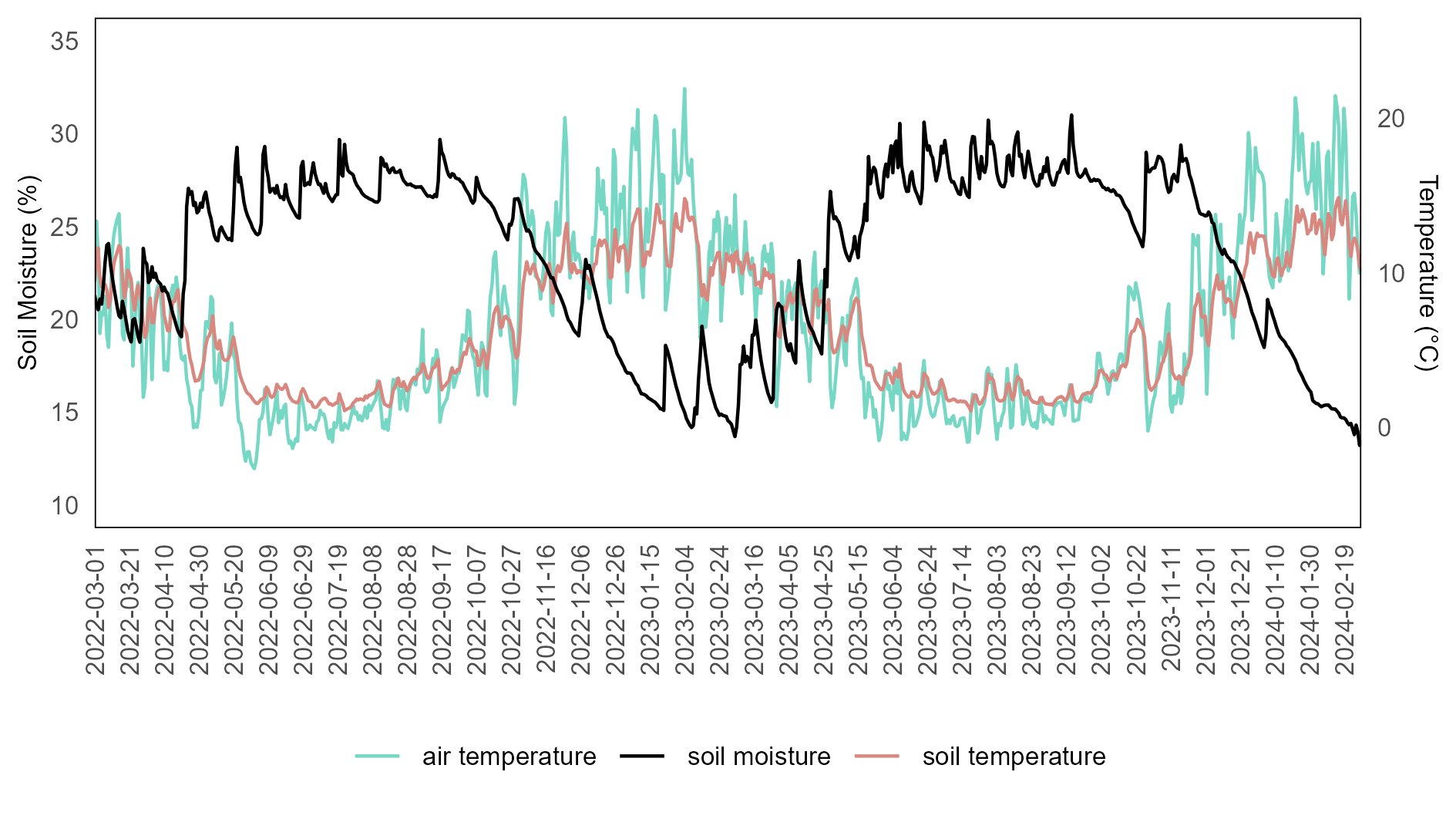


**Fig. 4** Mean daily temperature and mean daily soil moisture from all TMS loggers, where soil moisture and soil temperature are measured at a depth of -6 cm, and air temperature is measured at a height of 15 cm


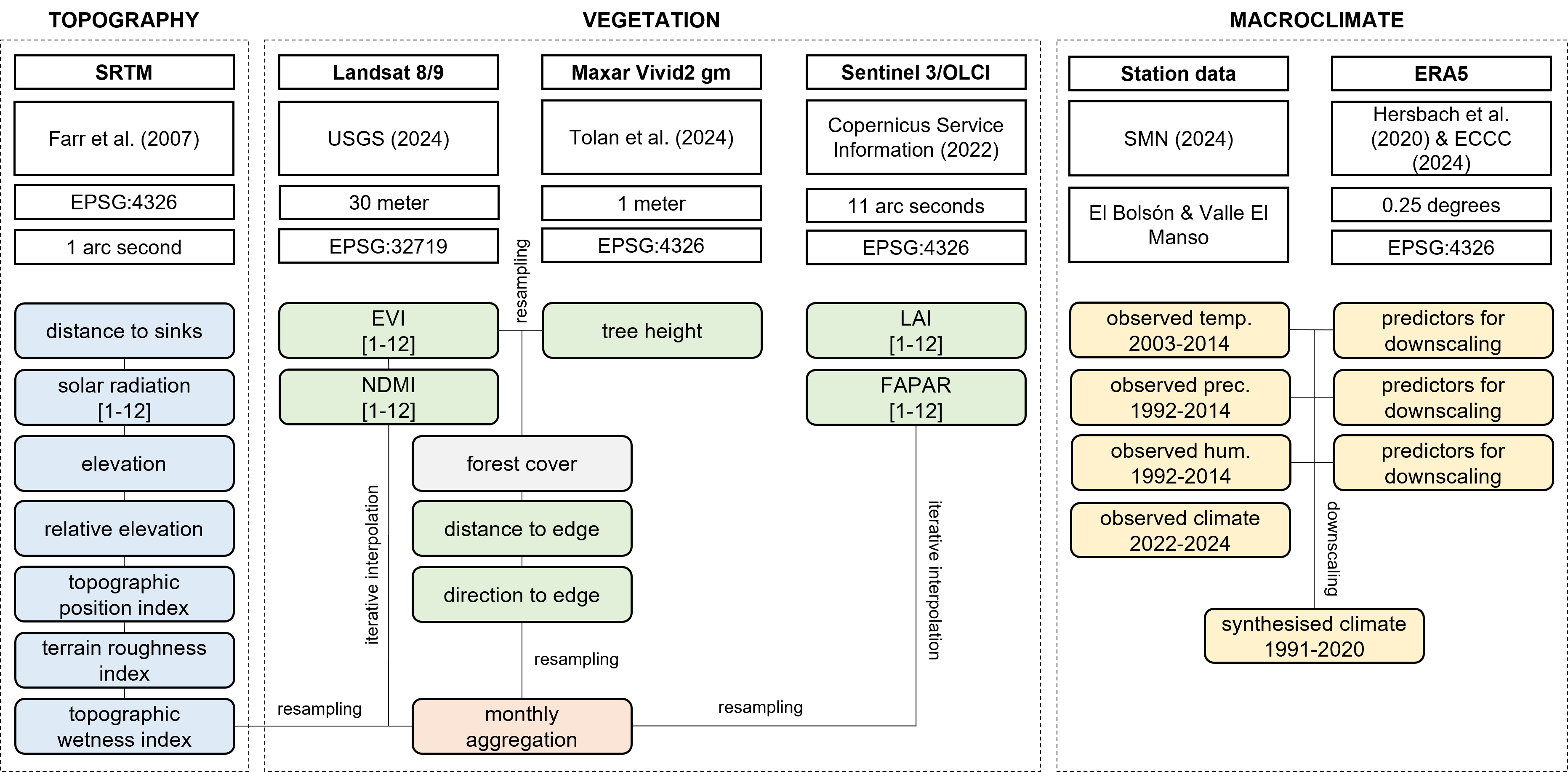


**Fig. 5** Biophysical predictors used for random forest-based regression model

**Table 1** Predictor variables for statistical downscaling of ERA5 data. Using even years from 2004 to 2014 for the Valle del Río Manso Inferior station and even years from 1991 to 2014 for the El Bolsón station, predictor variables were selected from the 26 available options (Table S1) based on their partial correlation coefficients (r) and p-values. Where hPa is the atmospheric pressure level in hectopascals

| **Variable (Station)** | **No.** | **ERA5 predictor variable** | **Partial r** | **P value** |
| --- | --- | --- | --- | --- |
| **TMAX: maximum daily temperature**  **(Valle del Río Manso Inferior)** | 1 | Mean sea level pressure | -0.242 | <0.0001 |
|  | 2 | 500 hPa Wind speed | -0.139 | <0.0001 |
|  | 3 | 500 hPa Meridional wind component | 0.184 | <0.0001 |
|  | 4 | 500 hPa Geopotential | 0.235 | <0.0001 |
|  | 5 | 850 hPa Zonal wind component | -0.120 | <0.0001 |
|  | 6 | 850 hPa Geopotential | 0.132 | <0.0001 |
| **TMIN: minimum daily temperature**  **(Valle del Río Manso Inferior)** | 1 | Mean sea level pressure | -0.195 | <0.0001 |
|  | 2 | 1000 hPa Wind speed | 0.261 | <0.0001 |
|  | 3 | 1000 hPa Relative vorticity of true wind | 0.190 | <0.0001 |
|  | 4 | 1000 hPa Divergence of true wind | -0.145 | <0.0001 |
|  | 5 | 500 hPa Geopotential | 0.290 | <0.0001 |
|  | 6 | Total precipitation | -0.112 | <0.0001 |
|  | 7 | 850 hPa Specific humidity | 0.313 | <0.0001 |
| **PRCP: daily sum of precipitation**  **(El Bolsón)** | 1 | 1000 hPa Zonal wind component | -0.189 | <0.0001 |
|  | 2 | 500 hPa Wind speed | 0.112 | <0.0001 |
|  | 3 | 850 hPa Wind Speed | 0.139 | <0.0001 |
|  | 4 | 850 hPa Geopotential | -0.240 | <0.0001 |
|  | 5 | 850 hPa Divergence of true wind | -0.107 | <0.0001 |
| **RH: relative humidity**  **(El Bolsón)** | 1 | 1000 hPa Divergence of true wind | 0.157 | <0.0001 |
|  | 2 | 850 hPa Divergence of true wind | -0.292 | <0.0001 |
|  | 3 | 850 hPa Specific humidity | 0.532 | <0.0001 |
|  | 4 | Air temperature at 2 m | -0.667 | <0.0001 |

**Table 2** Daily predictor variables for statistical downscaling as provided by (ECCC 2024)

| **No.** | **Variable ID** | **Predictor Variable** |
| --- | --- | --- |
| 1 | mslp | Mean sea level pressure |
| 2 | p1_f | 1000 hPa Wind speed |
| 3 | p1_u | 1000 hPa Zonal wind component |
| 4 | p1_v | 1000 hPa Meridional wind component |
| 5 | p1_z | 1000 hPa Relative vorticity of true wind |
| 6 | p1th | 1000 hPa Wind direction |
| 7 | p1zh | 1000 hPa Divergence of true wind |
| 8 | p5_f | 500 hPa Wind speed |
| 9 | p5_u | 500 hPa Zonal wind component |
| 10 | p5_v | 500 hPa Meridional wind component |
| 11 | p5_z | 500 hPa Relative vorticity of true wind |
| 12 | p5th | 500 hPa Wind direction |
| 13 | p5zh | 500 hPa Divergence of true wind |
| 14 | p8_f | 850 hPa Wind Speed |
| 15 | p8_u | 850 hPa Zonal wind component |
| 16 | p8_v | 850 hPa Meridional wind component |
| 17 | p8_z | 850 hPa Relative vorticity of true wind |
| 18 | p8th | 850 hPa Wind direction |
| 19 | p8zh | 850 hPa Divergence of true wind |
| 20 | p500 | 500 hPa Geopotential |
| 21 | p850 | 850 hPa Geopotential |
| 22 | prcp | Total precipitation |
| 23 | s500 | 500 hPa Specific humidity |
| 24 | s850 | 850 hPa Specific humidity |
| 25 | shum | 1000 hPa Specific humidity |
| 26 | temp | Air temperature at 2 m |


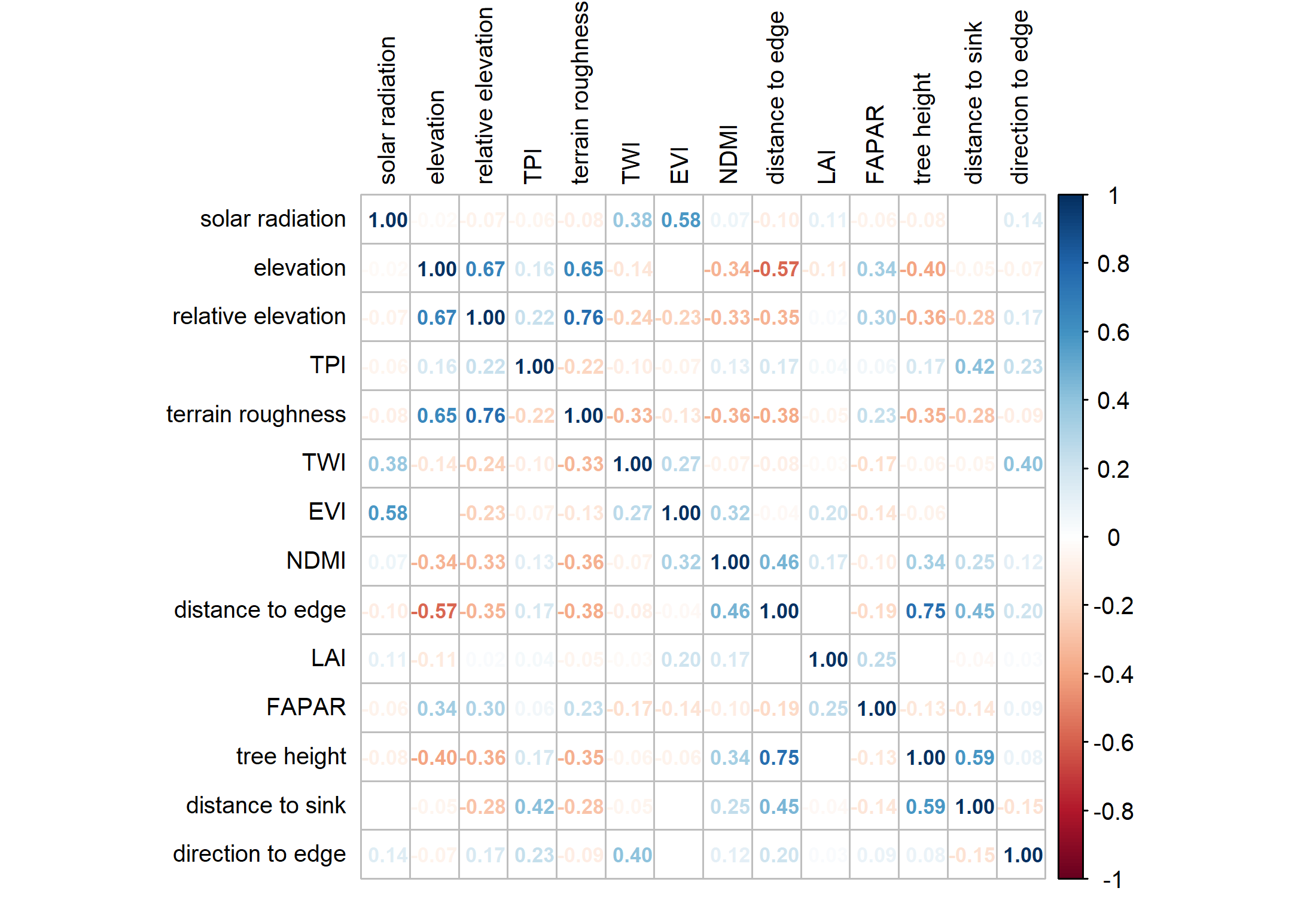


**Fig. 6** Correlation matrix for the identification of variable to be excluded. Significance level at 0.01. Variables with significant correlation higher than ±0.7 were excluded. Visualisation using *corrplot* package in Rstudio (Wei and Simko 2010)

**Table 3** Model performance over all months

| **Model** | **R²** | **R² SD** | **RMSE** | **RMSE SD** | **MAE** | **MAE SD** |
| --- | --- | --- | --- | --- | --- | --- |
| **TMIN 2 m** | 0.73 | 0.03 | 1.76 | 0.11 | 1.37 | 0.09 |
| **TMIN 15 cm** | 0.74 | 0.04 | 1.40 | 0.10 | 1.03 | 0.06 |
| **VWC -6 cm** | 0.77 | 0.03 | 3.56 | 0.22 | 2.72 | 0.18 |
| **TMIN -6 cm** | 0.78 | 0.03 | 1.17 | 0.08 | 0.89 | 0.06 |
| **RH 2 m** | 0.79 | 0.04 | 6.72 | 0.81 | 4.66 | 0.46 |
| **TMAX -6 cm** | 0.85 | 0.02 | 1.11 | 0.07 | 0.82 | 0.05 |
| **TMAX 15 cm** | 0.85 | 0.03 | 2.38 | 0.24 | 1.64 | 0.14 |
| **TMAX 2 m** | 0.88 | 0.02 | 1.46 | 0.12 | 1.11 | 0.08 |


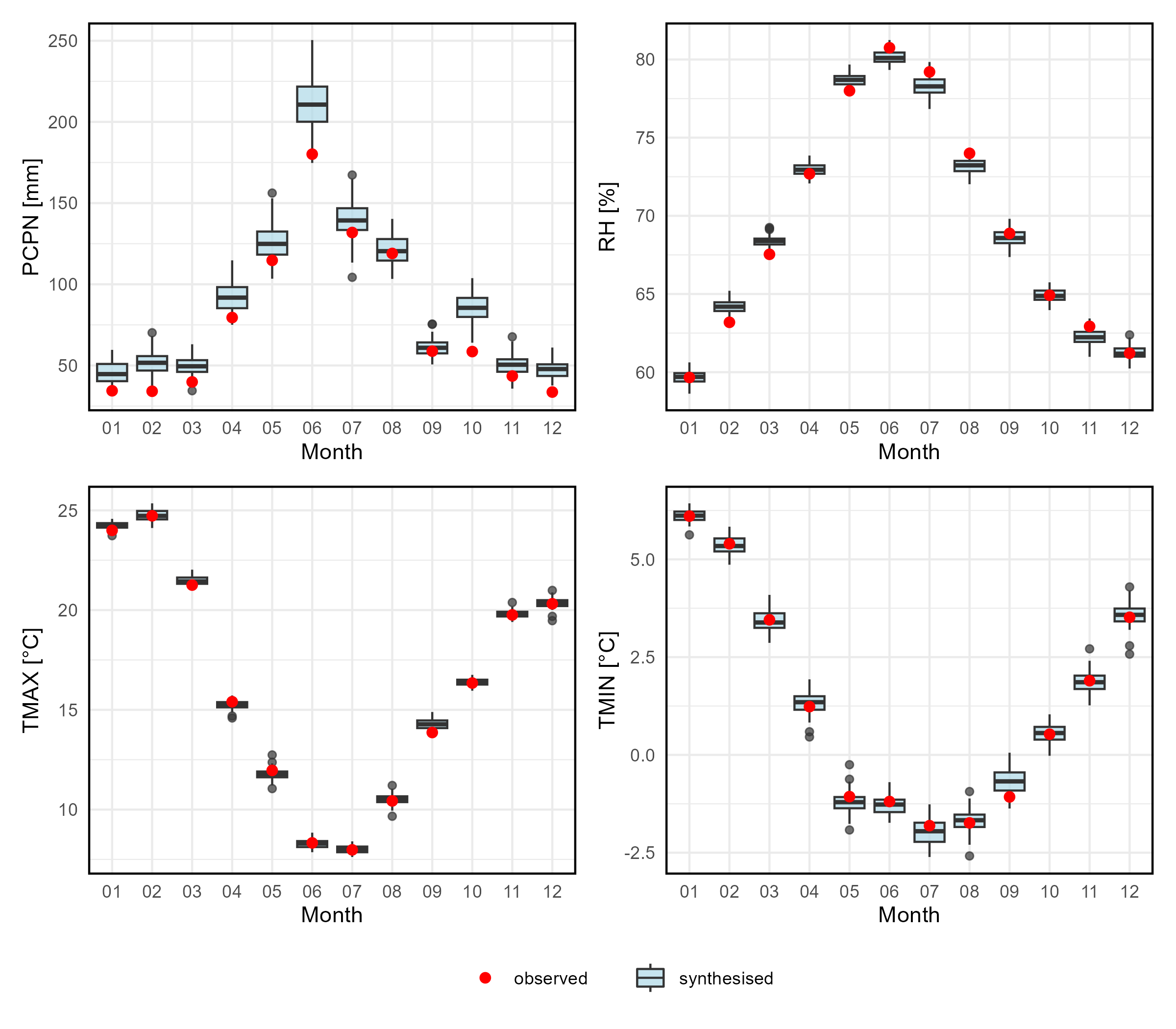


**Fig. 7** Validation results of statistical downscaling. Based on weather station data of Valle El Manso (TMAX and TMIN) as well as El Bolsón (PCPN and RH)


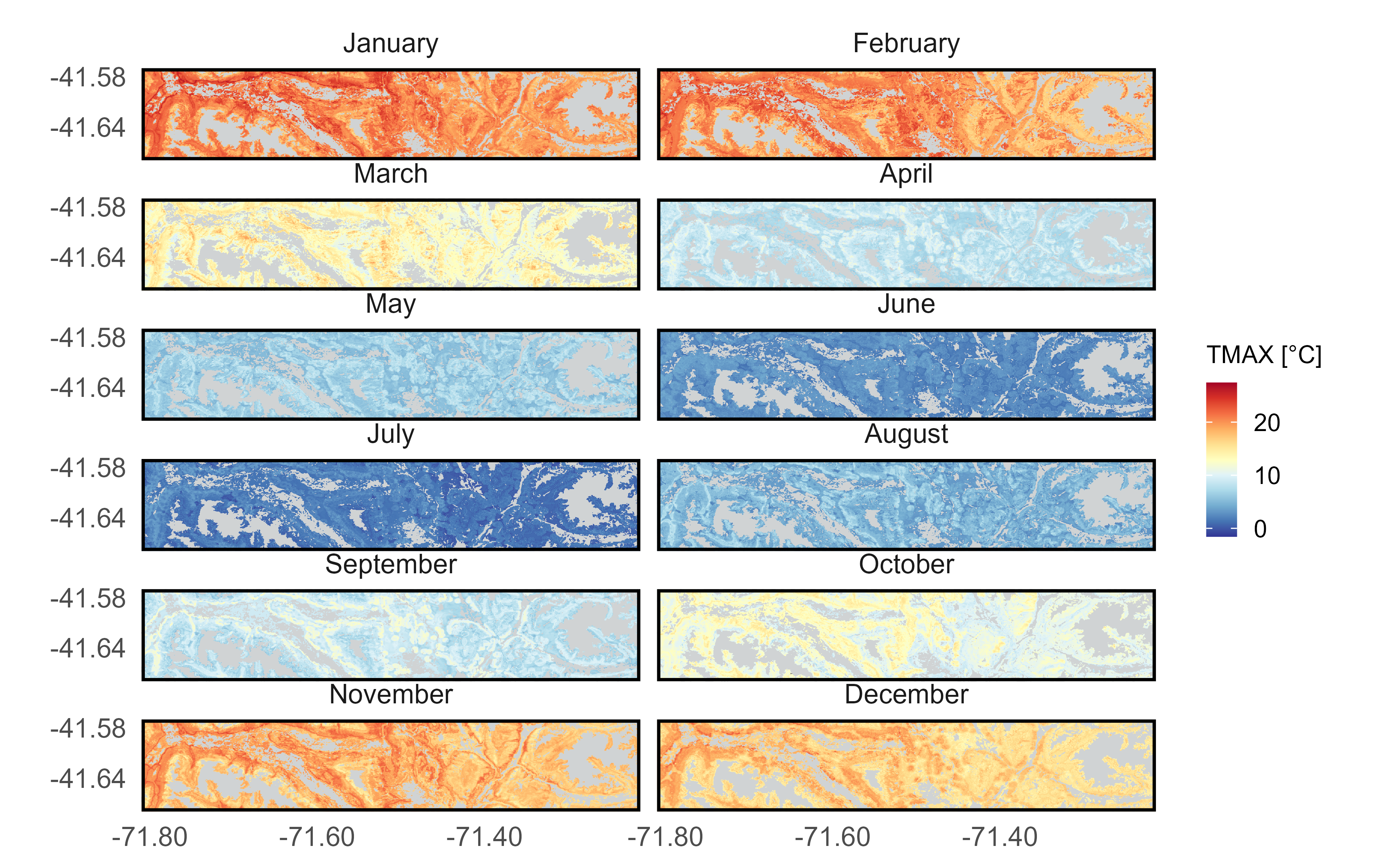


**Fig. 8** Maximum temperature at 2m height between 2022-2023


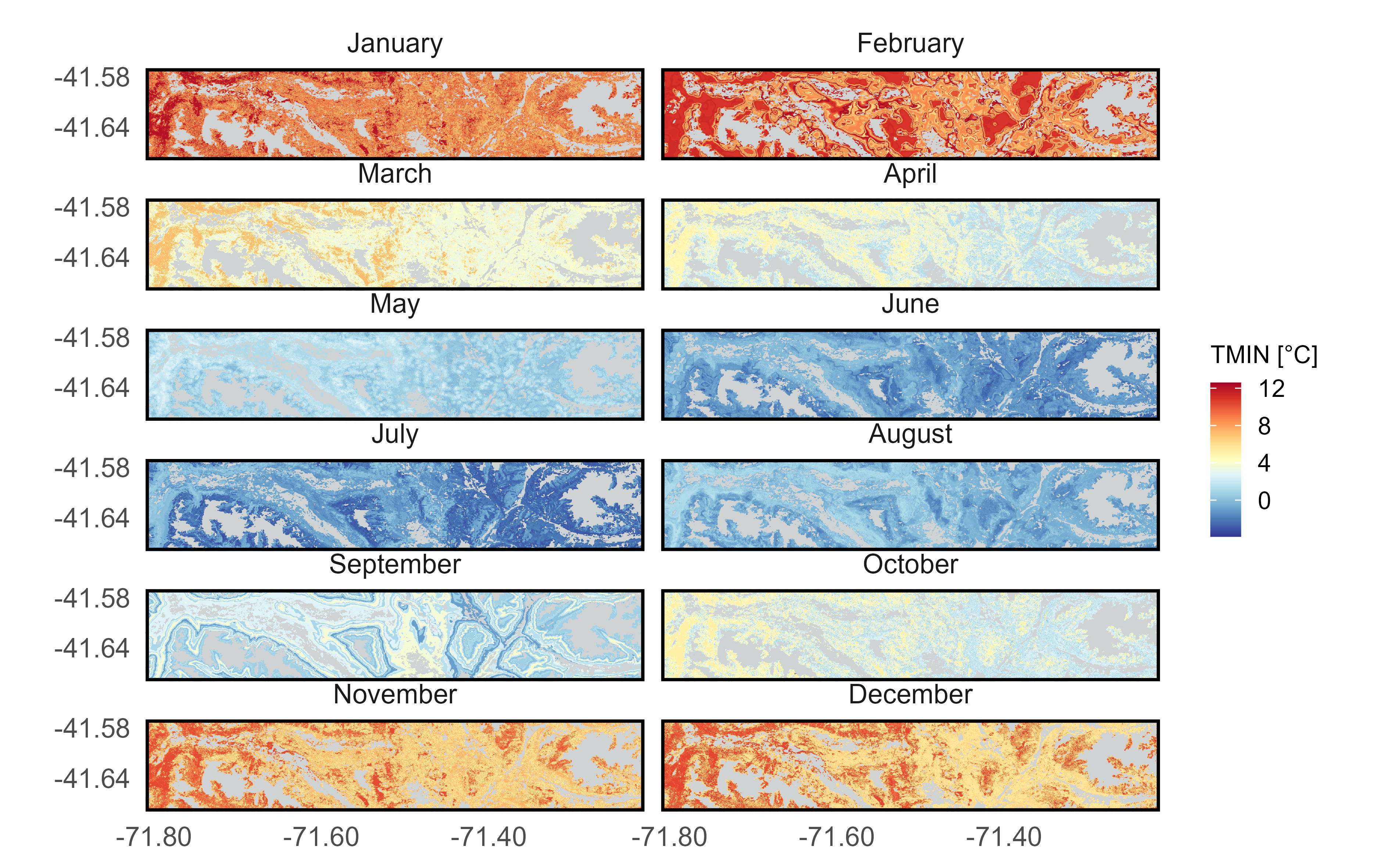


**Fig. 9** Minimum temperature at 2m height between 2022-2023


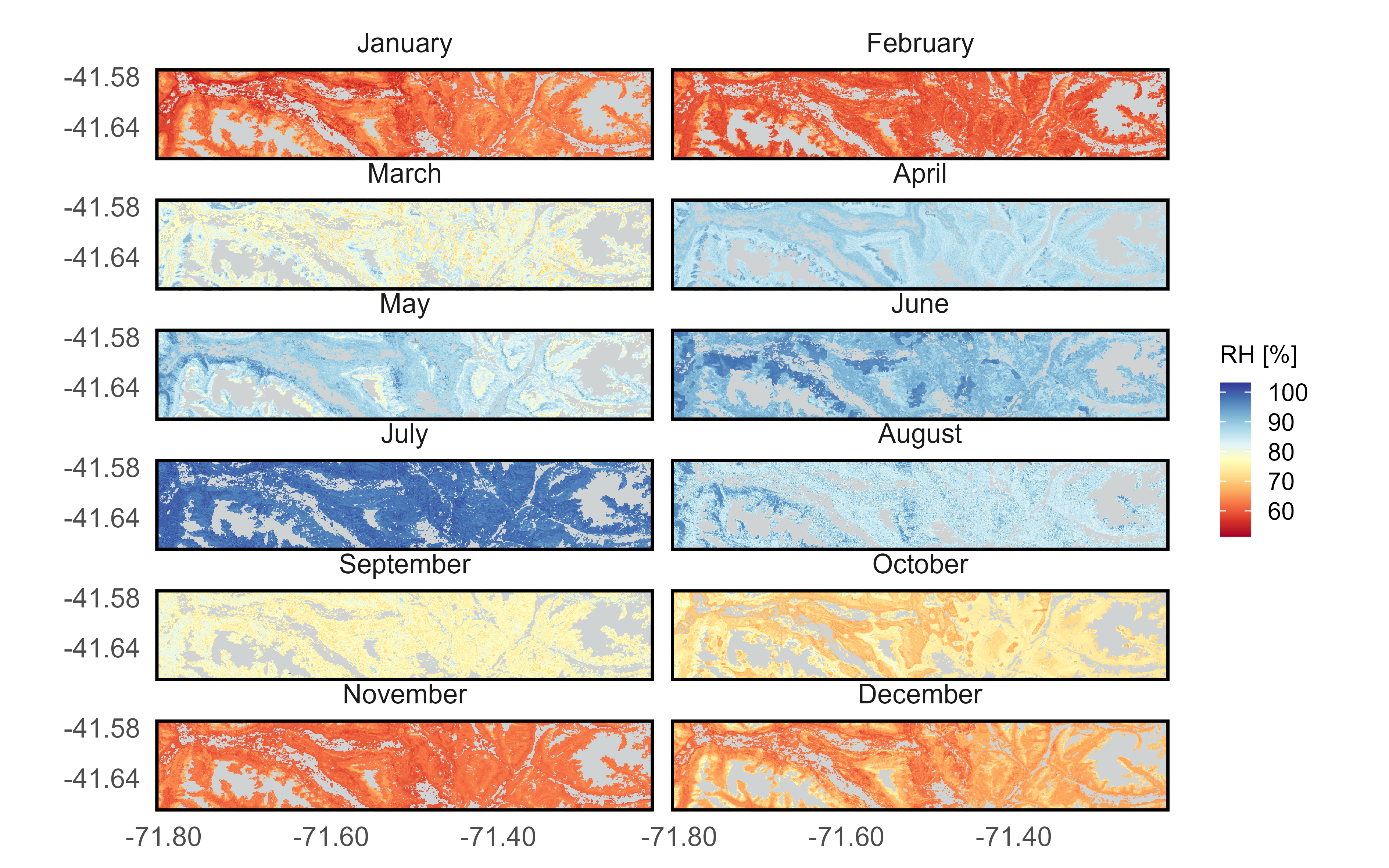


**Fig. 10** Relative humidity at 2m height between 2022-2023


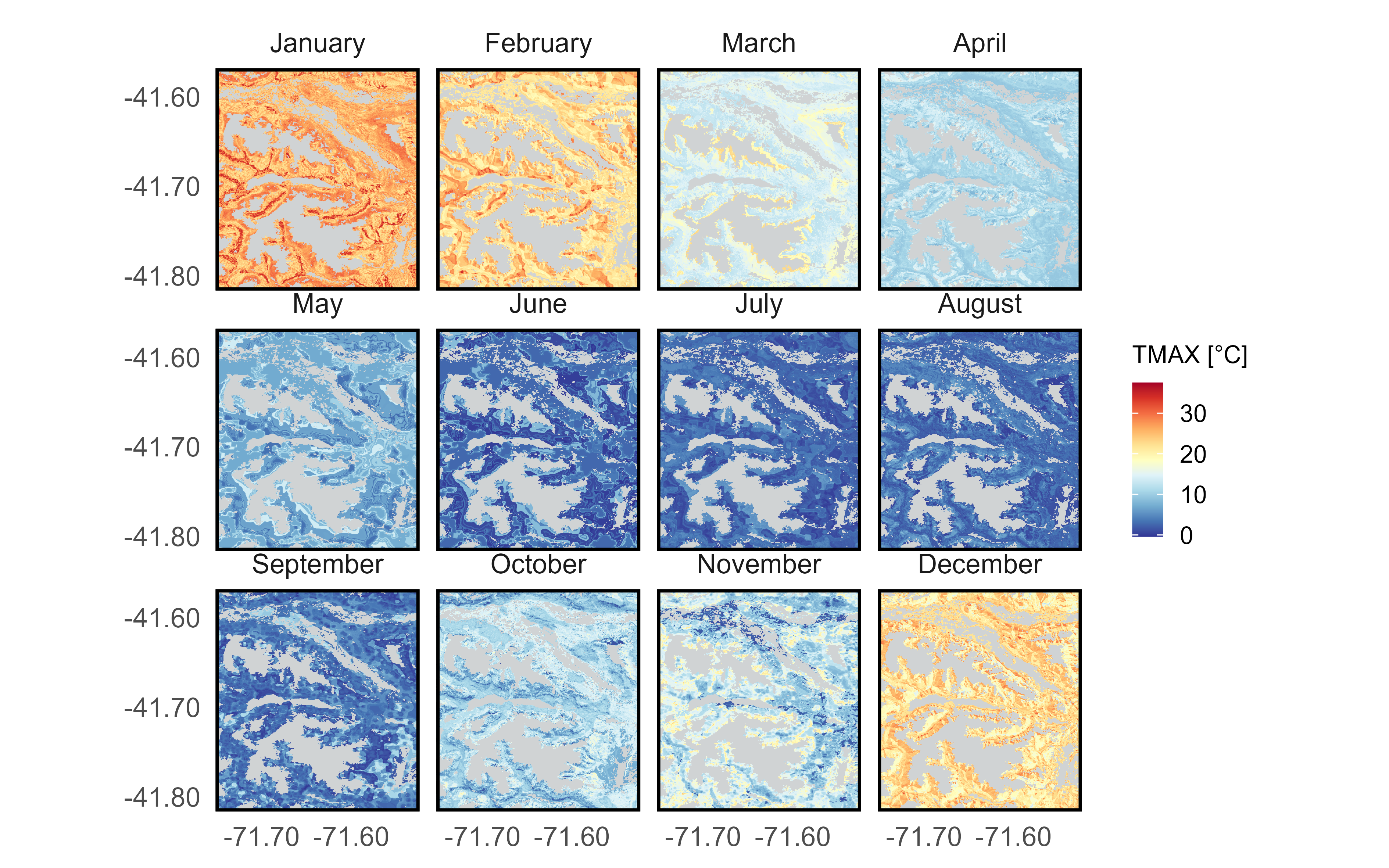


**Fig. 11** Maximum temperature at 15 cm between 2022-2024


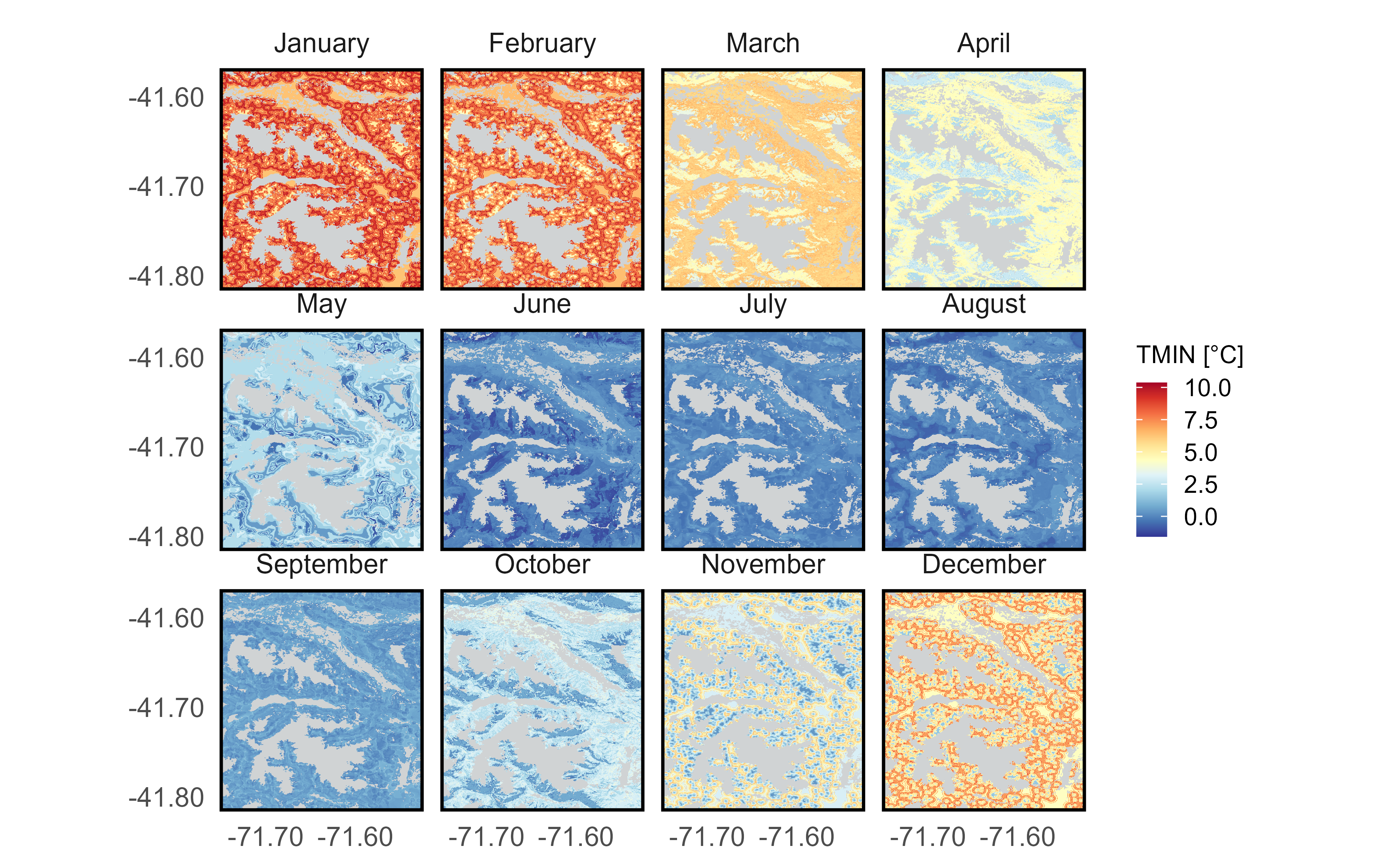


**Fig. 12** Minimum temperature at 15 cm between 2022-2024


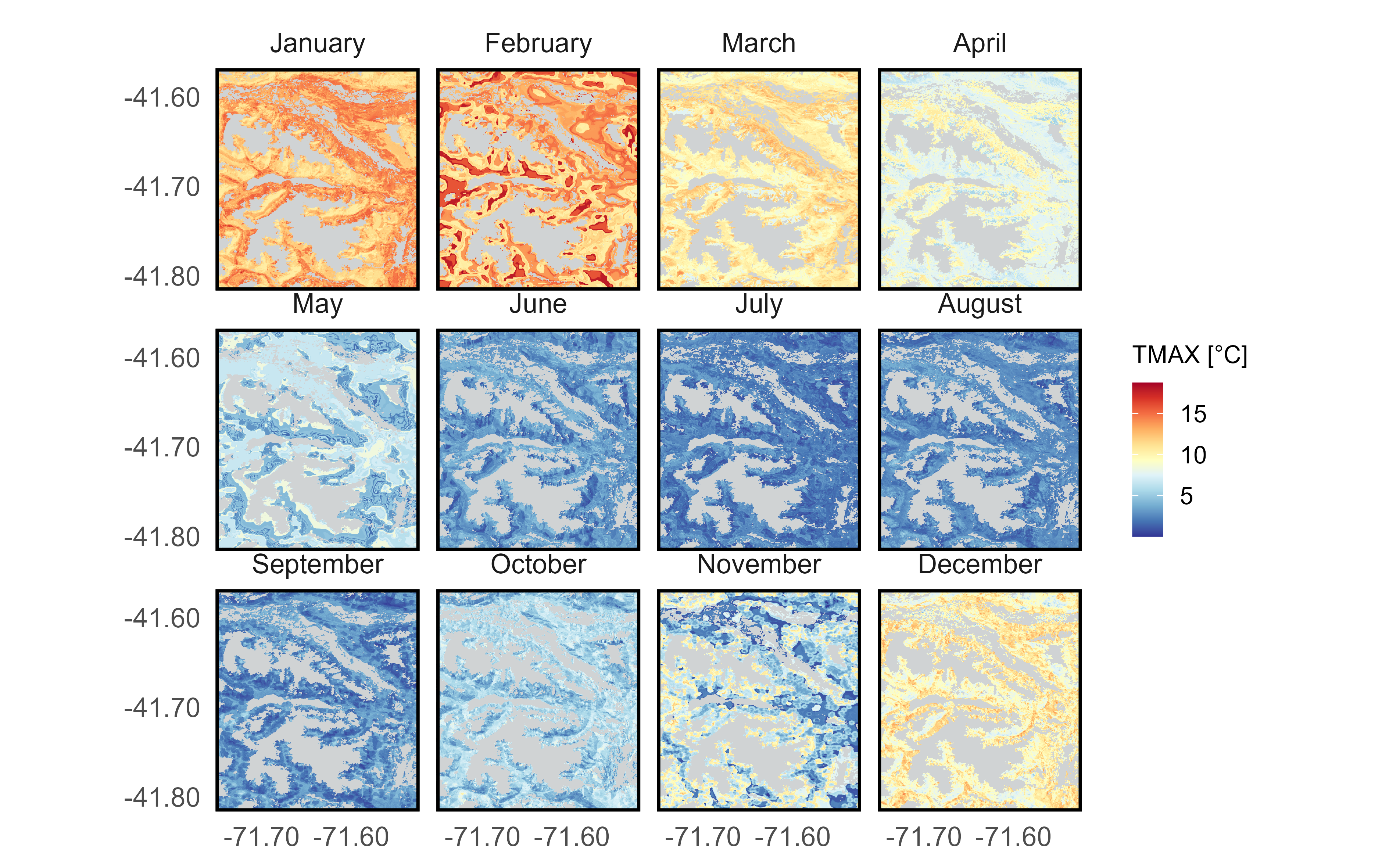


**Fig. 13** Maximum temperature at -6 cm depth between 2022-2024


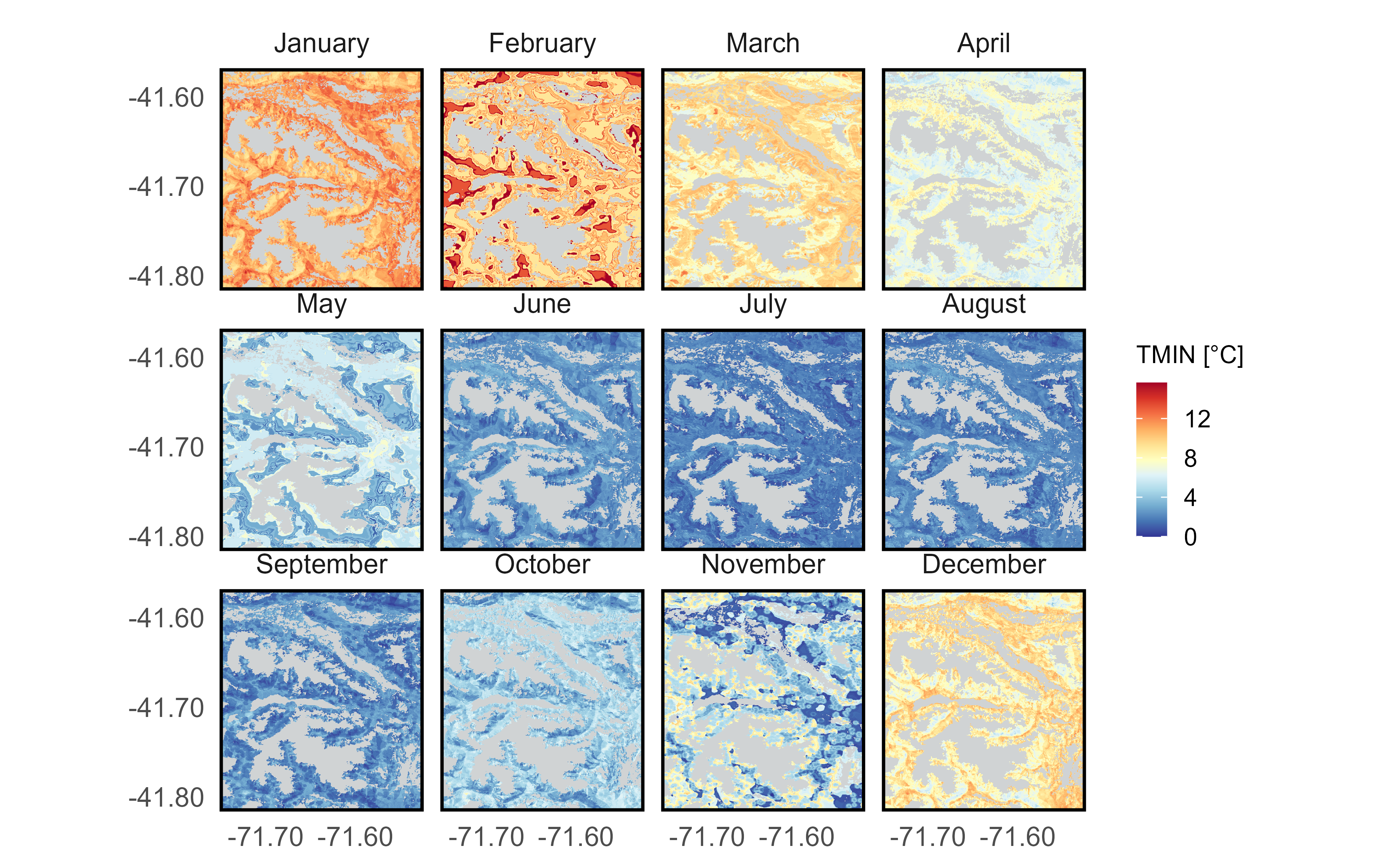


**Fig. 14** Minimum temperature at -6 cm depth between 2022-2024


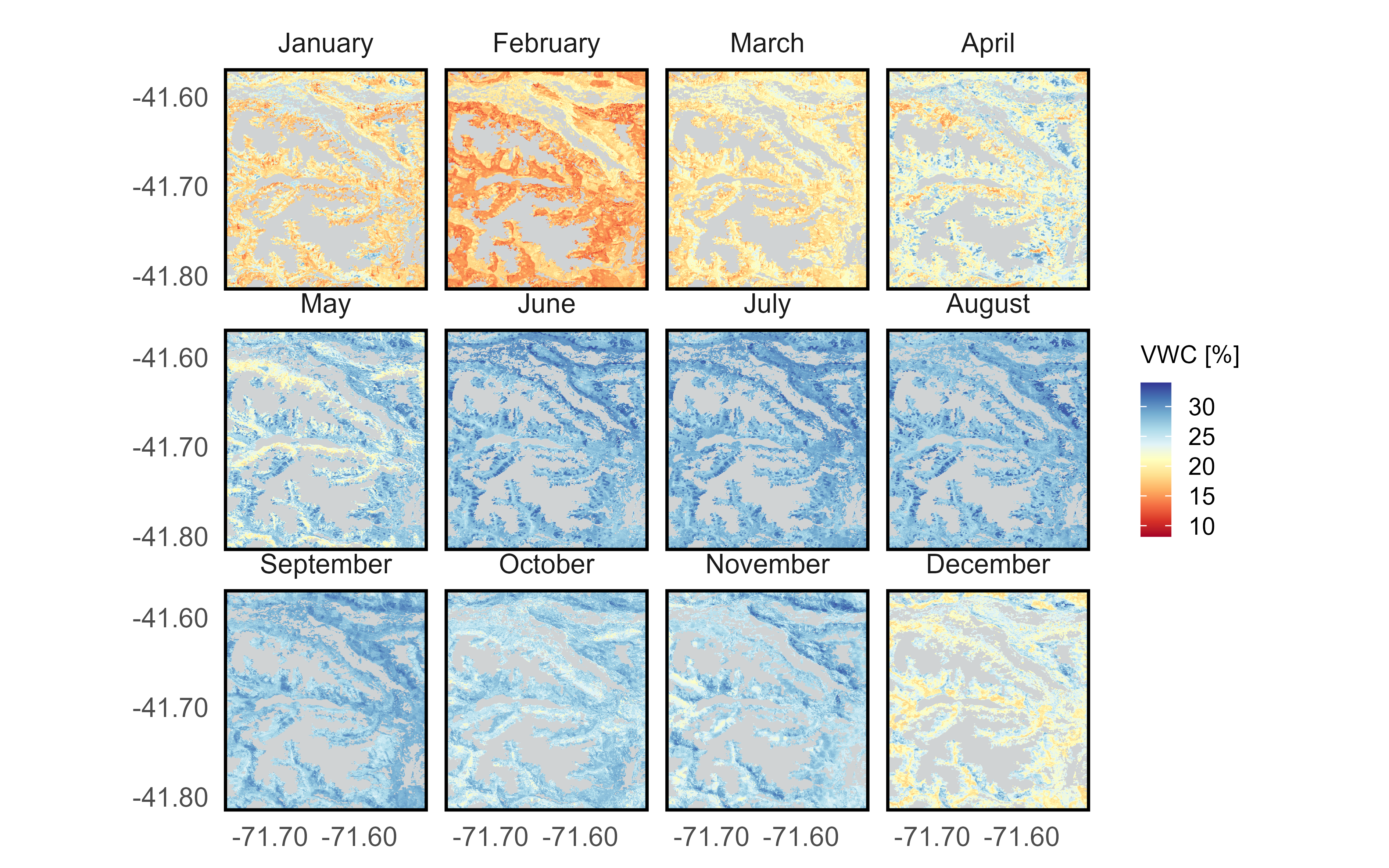


**Fig. 15** VWC at -6 cm depth between 2022-2024


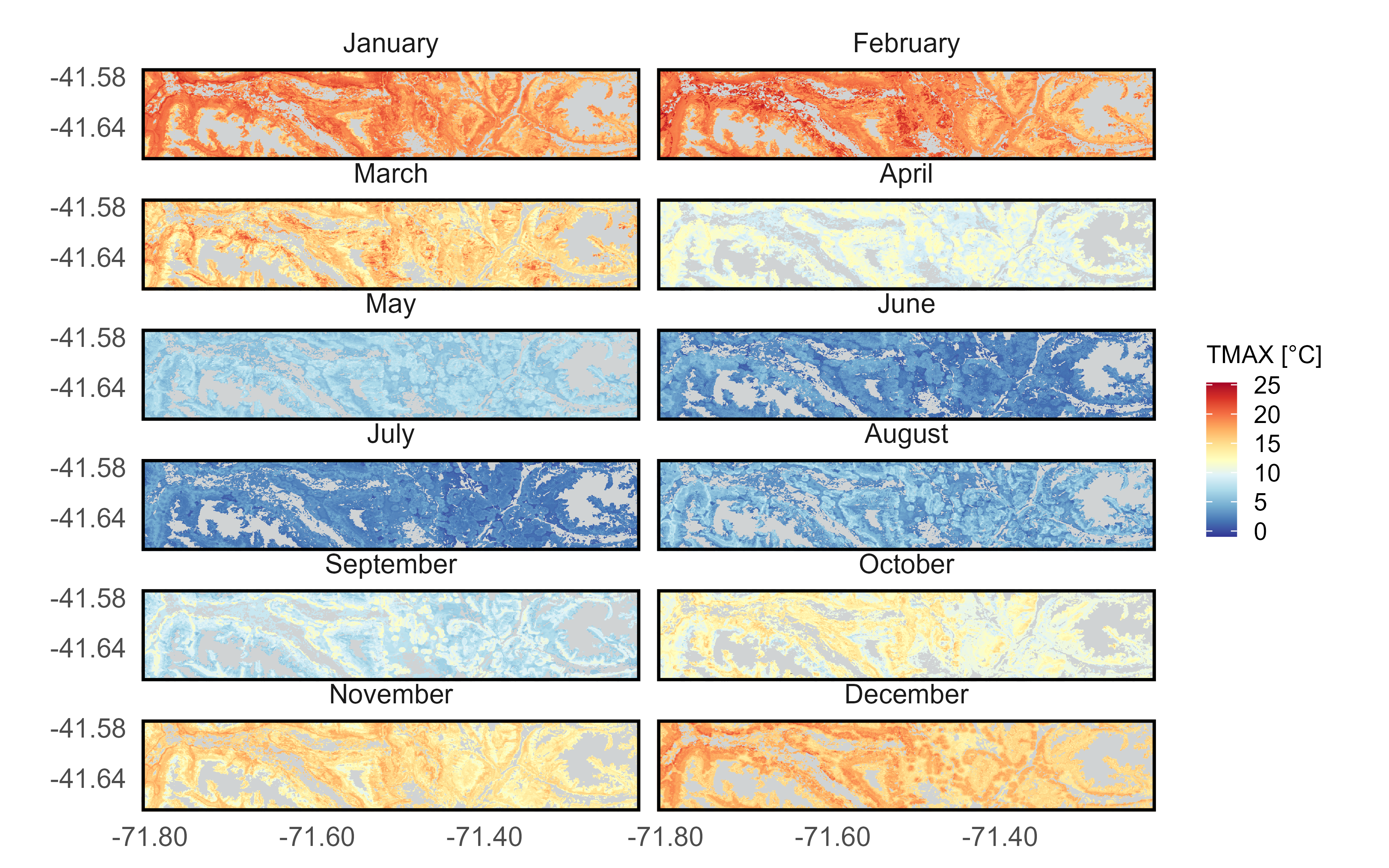


**Fig. 16** Maximum temperature at 2m height between 1981-2010


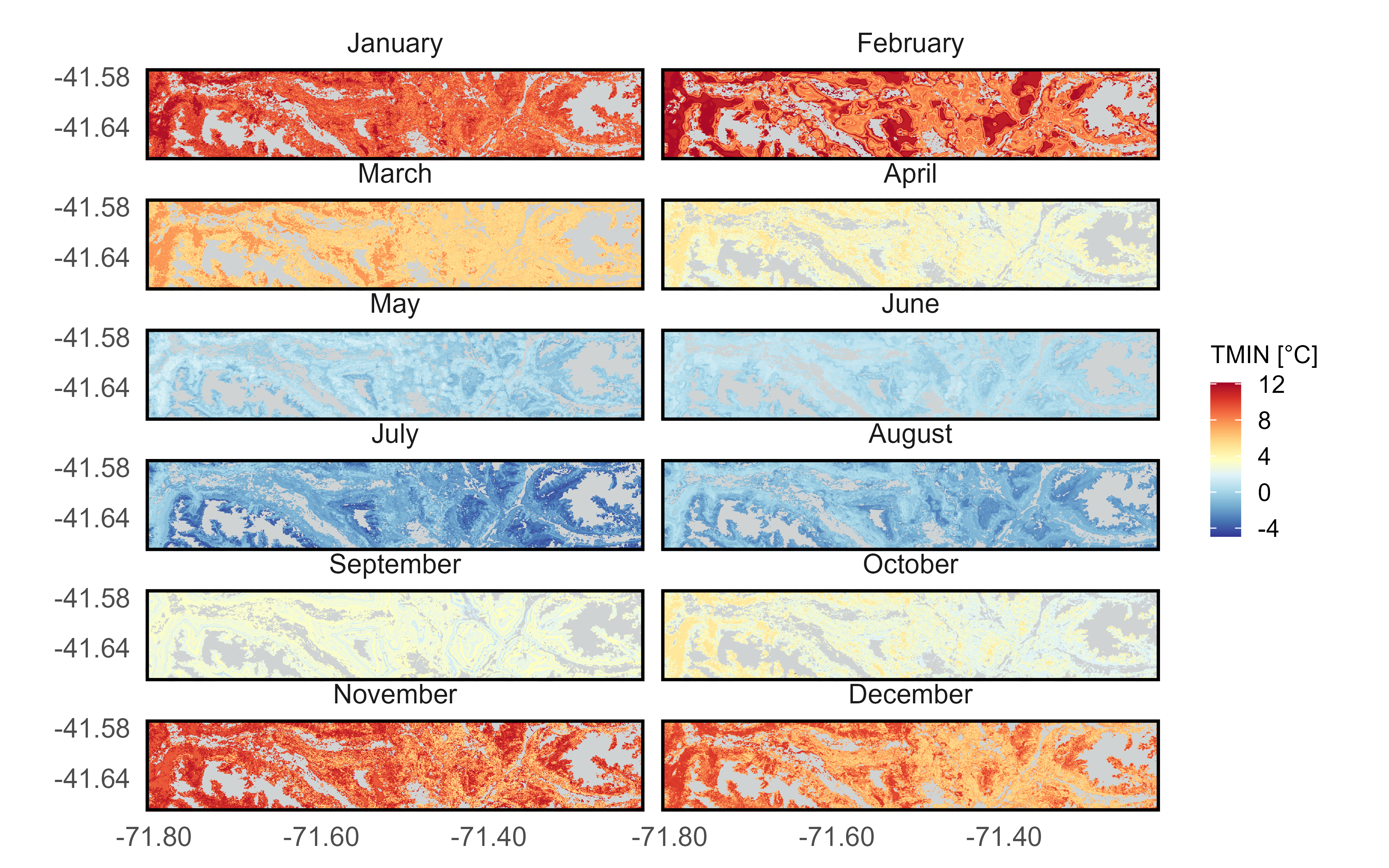


**Fig. 17** Minimum temperature at 2m height between 1981-2010


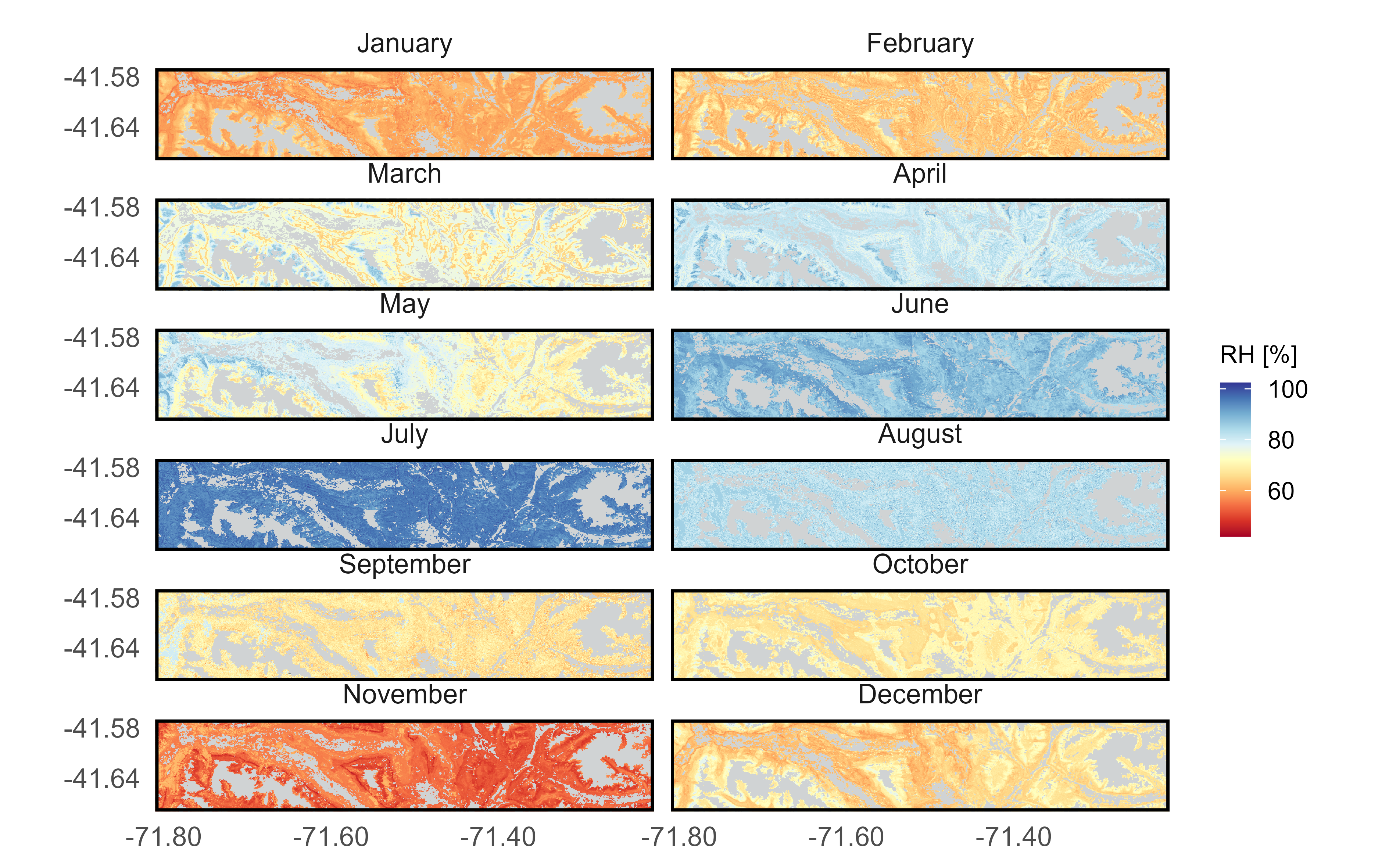


**Fig. 18** Relative humidity at 2m height between 1981-2010


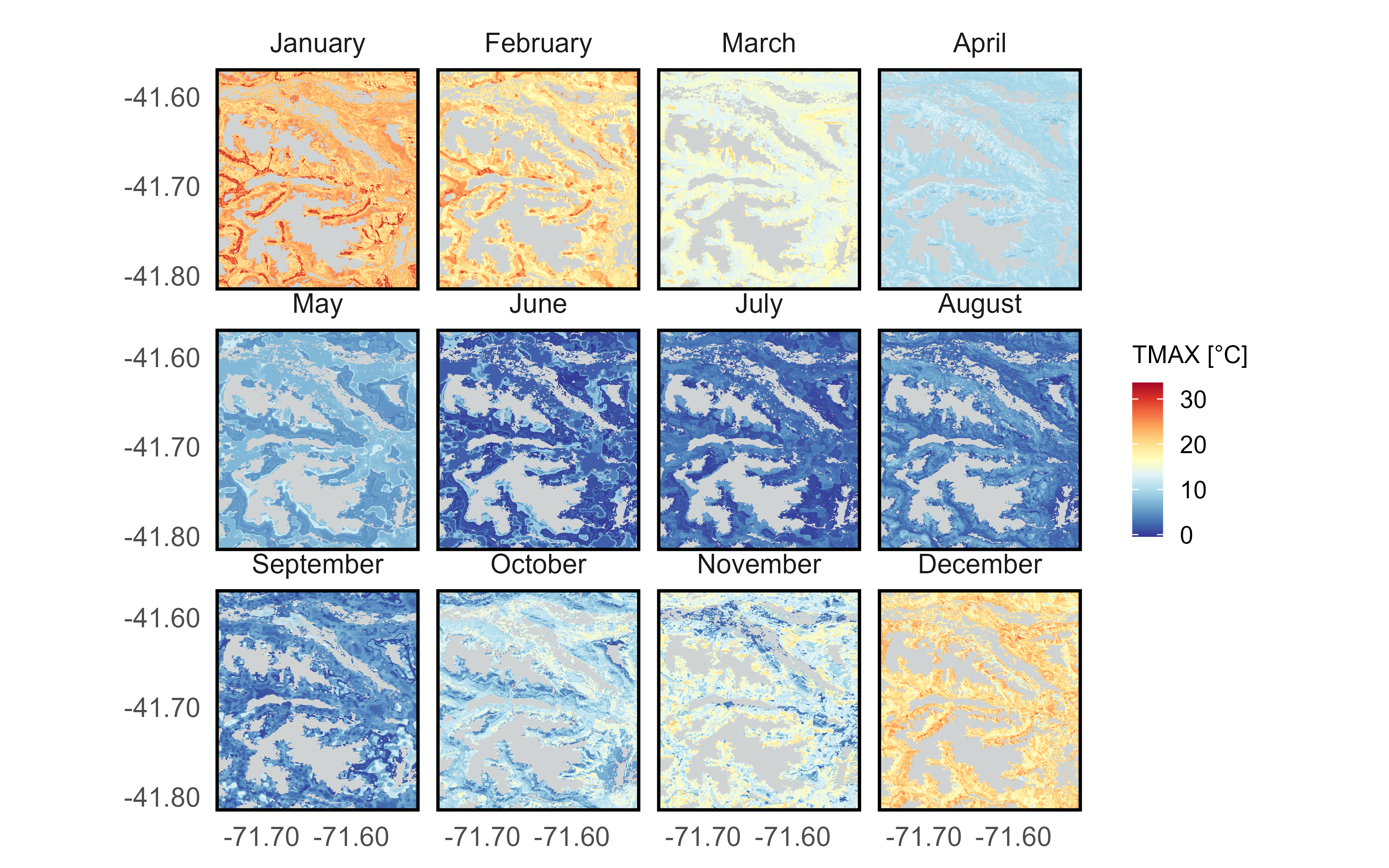


**SF 19** Maximum temperature at 15 cm height between 1981-2010


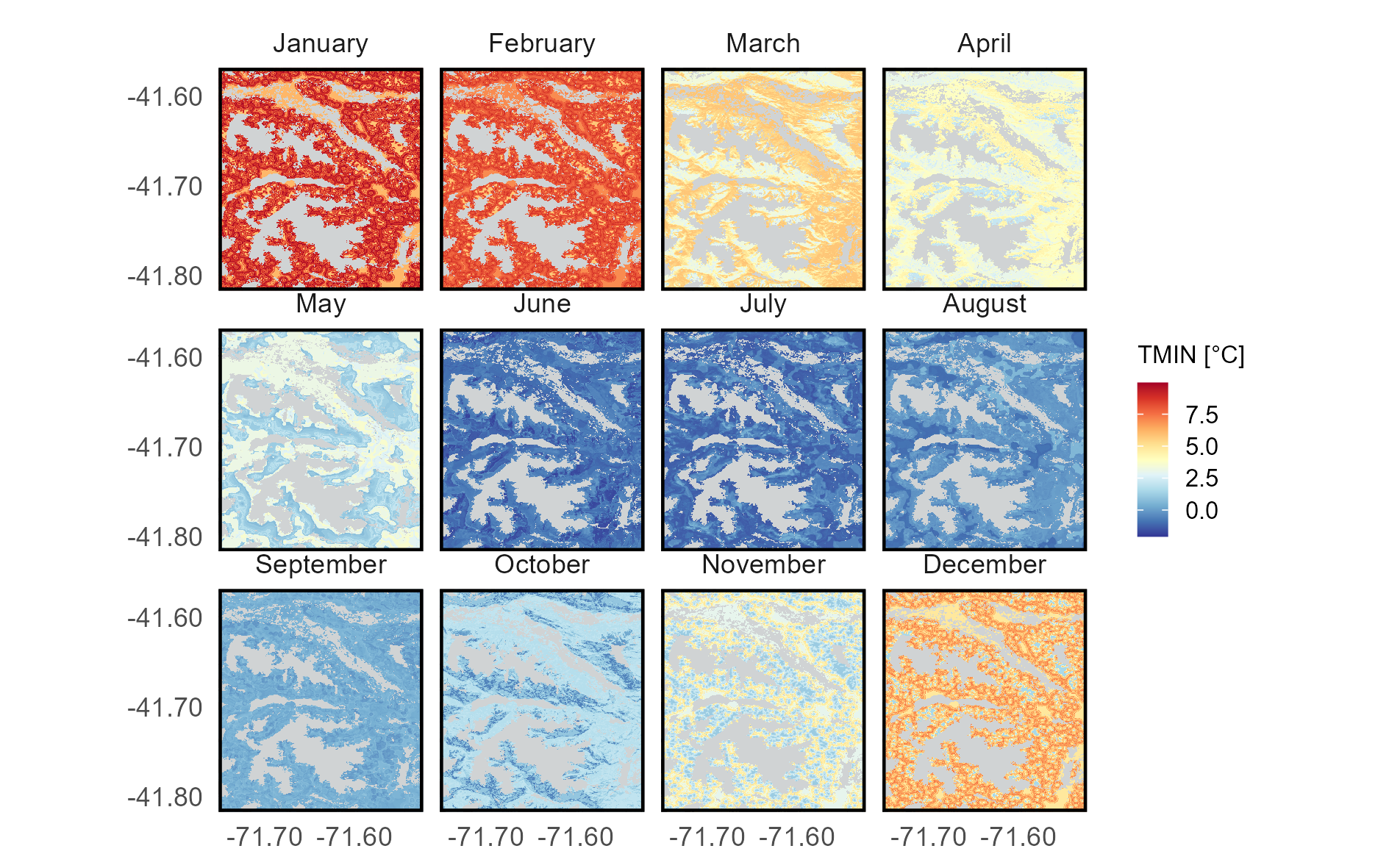


**Fig. 20** Minimum temperature at 15 cm height between 1981-2010


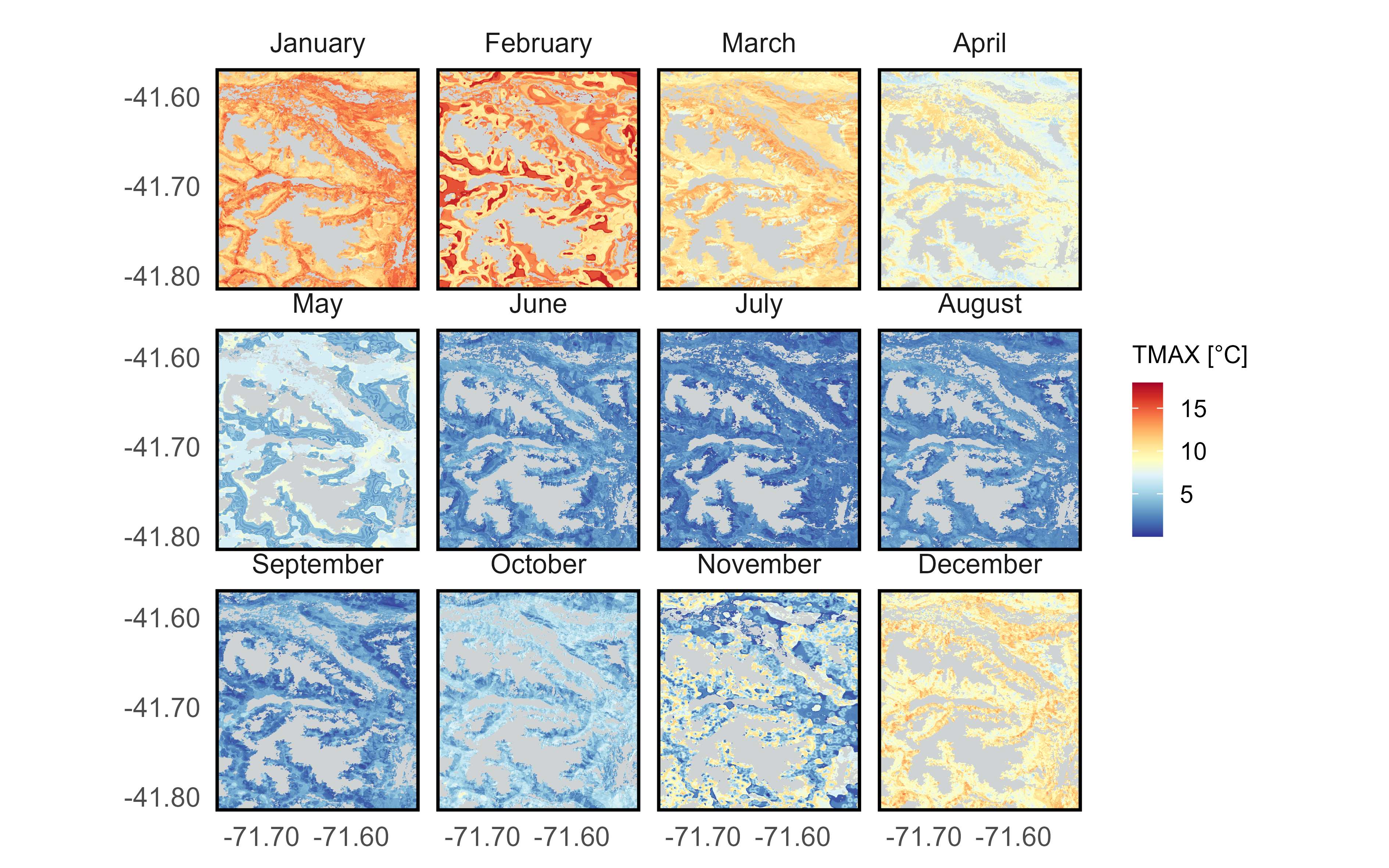


**Fig. 21** Maximum temperature at -6 cm depth between 1981-2010


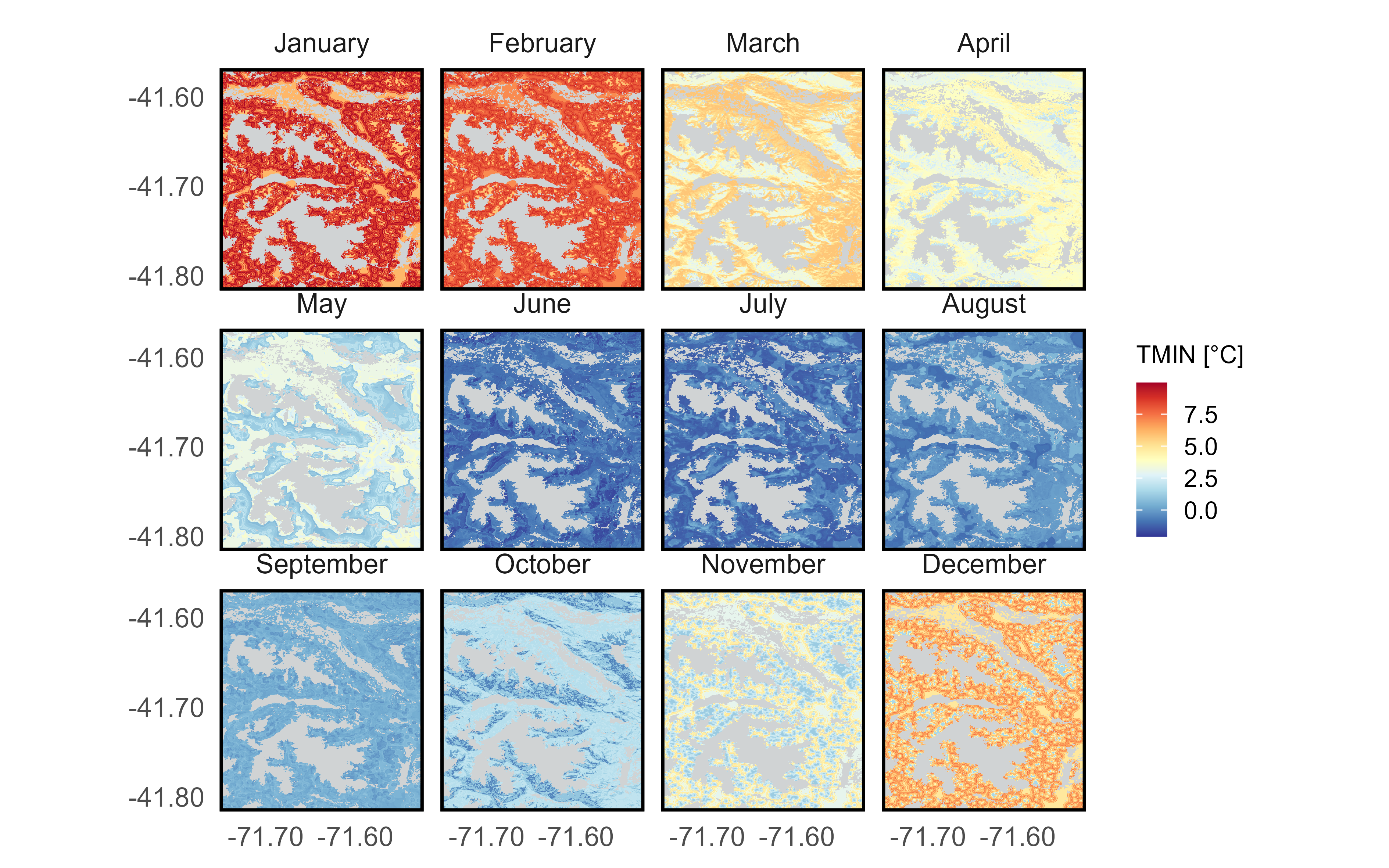


**Fig. 22** Minimum temperature at -6 cm depth between 1981-2010


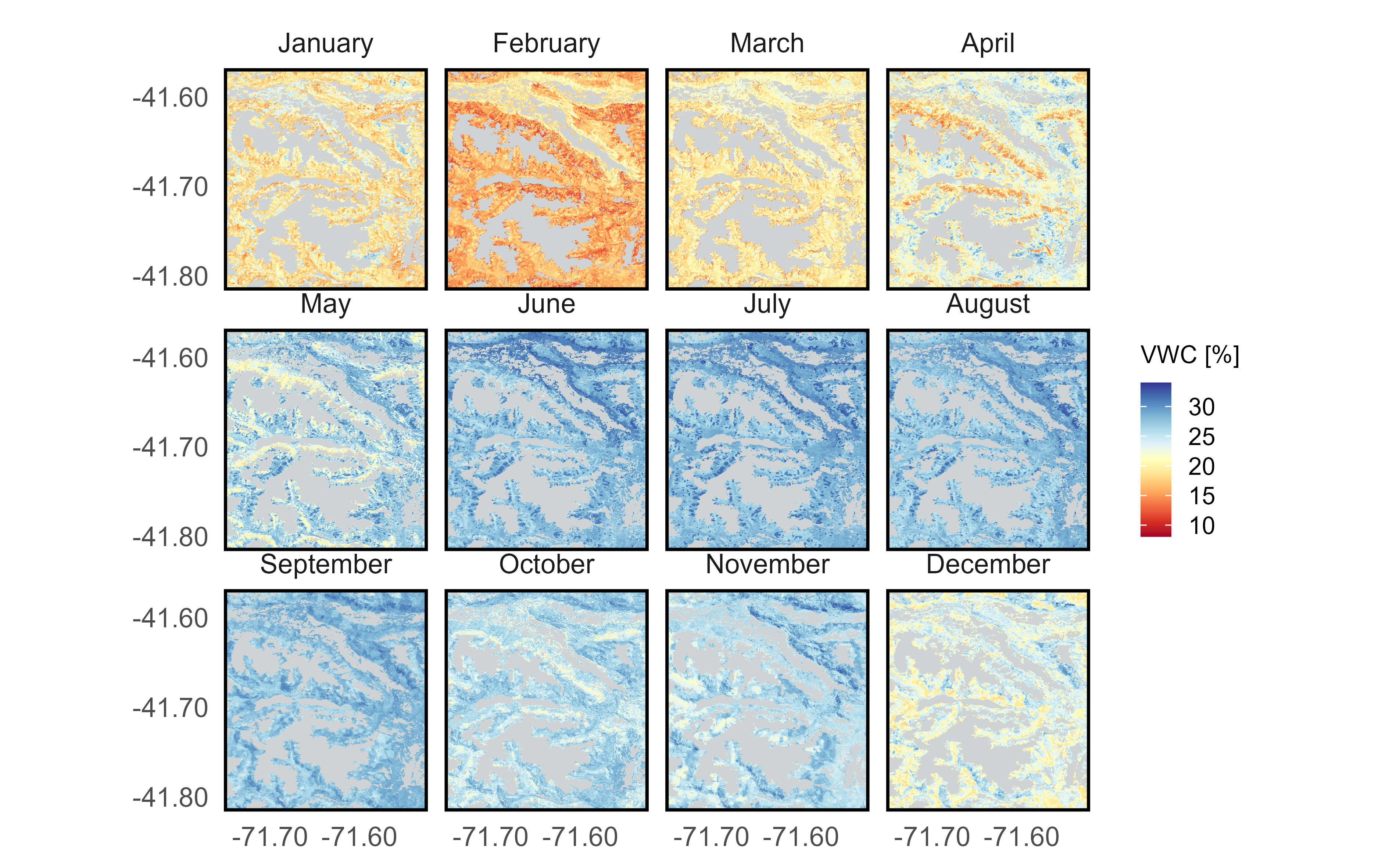


**Fig. 23** VWC at -6 cm depth between 1981-2010

References

ECCC (2024) CMIP6 ensemble of daily predictor variables. <https://climate-scenarios.canada.ca/?page=pred-cmip6#table-1>. Accessed 15 April 2024

Wei T, Simko V (2010) corrplot: Visualization of a Correlation Matrix. CRAN: Contributed Packages. The R Foundation. <https://doi.org/10.32614/CRAN.package.corrplot>
